# The Role of the Intestinal Microbiome in Multiple Sclerosis—Lessons to Be Learned from Hippocrates

**DOI:** 10.3390/biology12121463

**Published:** 2023-11-24

**Authors:** Mohamed Mahmoud El-Sayed, Sidhesh Mohak, Dhir Gala, Reka Fabian, Zoltan Peterfi, Zsolt Fabian

**Affiliations:** 1 School of Medicine and Dentistry, Faculty of Clinical and Biomedical Sciences, University of Central Lancashire, Fylde Rd, Preston PR1 2HE, UK; mmelsayed@uclan.ac.uk; 2 Department of Clinical Sciences, Saint James School of Medicine, Park Ridge, IL 60068, USA; smohak@mail.sjsm.org; 3 American University of the Caribbean School of Medicine, 1 University Drive, Jordan Road, Cupecoy, St Marteen, The Netherlands; dhirgala@students.aucmed.edu; 4 Salerno, Secondary School, Threadneedle Road, H91 D9H3 Galway, Ireland; rekafabian2024@salerno.ie; 5 Division of Infectology, 1st Department of Internal Medicine, University of Pecs, Clinical Centre, 7623 Pécs, Hungary; peterfi.zoltan@pte.hu

**Keywords:** intestinal microbiome, multiple sclerosis, macrophages, regulatory T lymphocytes

## Abstract

**Simple Summary:**

Multiple sclerosis is one of the long-term human diseases that significantly affects quality of life through the decline of muscle strength, problems in the coordination of muscle functions leading to disability or the decline of visual and cognitive functions. Although extensive research in the field of multiple sclerosis identified the abnormal function of the patients’ immune systems as the primary mechanism underlying the disease, the ultimate cause of the autoimmune nature of multiple sclerosis, remained unknown. Recent research, however, shed light on the possible role of the bacteria living in the human gut, termed the human intestinal microbiome, in the development of multiple sclerosis. In this article, we review our current knowledge on the interplay between the intestinal microbiome and the immune mechanisms involved in the development of multiple sclerosis.

**Abstract:**

Based on recent advances in research of chronic inflammatory conditions, there is a growing body of evidence that suggests a close correlation between the microbiota of the gastrointestinal tract and the physiologic activity of the immune system. This raises the idea that disturbances of the GI ecosystem contribute to the unfolding of chronic diseases including neurodegenerative pathologies. Here, we overview our current understanding on the putative interaction between the gut microbiota and the immune system from the aspect of multiple sclerosis, one of the autoimmune conditions accompanied by severe chronic neuroinflammation that affects millions of people worldwide.

## 1. Introduction

Chronic inflammatory human diseases present a growing medical challenge to the healthcare systems in developed countries [1]. Despite the diversity of the involved tissues and organs, one of the possible underlying mechanisms is the disturbed control of the immune system that develops autoimmunity. Although the molecular details are still not clear, recent research suggests that the microbiota of the gastrointestinal (GI) tract plays an important role in the physiologic activity of the immune system, raising the idea that disturbances of the GI ecosystem contribute to the unfolding of autoimmune conditions. Here, we overview this putative interaction in the context of multiple sclerosis (MS), one of the autoimmune conditions accompanied by severe chronic neuroinflammation affecting millions of people worldwide.

## 2. Multiple Sclerosis

Multiple sclerosis (MS) is a common multifactorial chronic neurodegenerative disorder involving the central nervous system (CNS) affecting around 2 million people worldwide [2,3]. The primary symptoms of MS are directly related to the demyelination of axons, consisting sensory issues and the impairment of motor functions like ataxia and weakness [4]. Primary symptoms can lead to secondary complications including recurrent urinary tract infections due to impaired neuromuscular functions of the bladder or psychological problems like depression [5,6]. Based on the initial presenting symptoms, the disease courses are grouped into four major classes including the relapsing–remitting, primary progressive, secondary progressive and clinically isolated forms [7]. Currently, there is no cure available for MS, so mainstream therapeutic approaches focus on modifying disease progression by reducing the number of flareups and treating individual symptoms to improve patients’ life quality [8].

**Figure 1:**
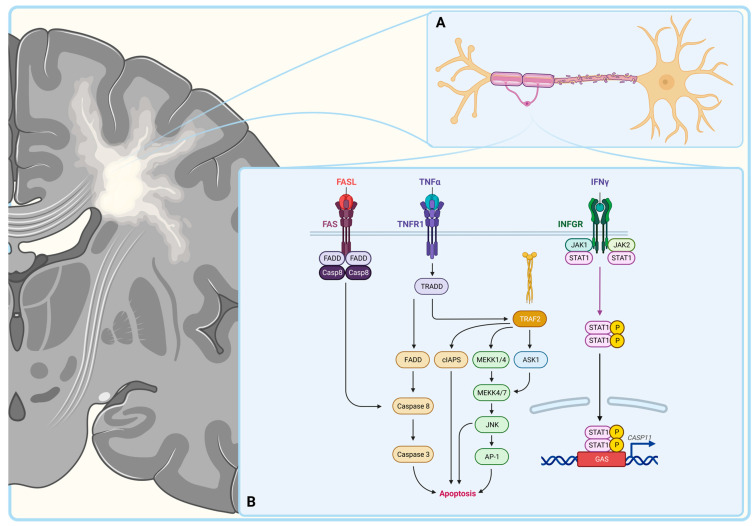
Pathological hallmark of multiple sclerosis. Multiple sclerosis is one of the demyelinating disorders with the leading pathological finding being the destruction of the myelin sheet around the axons (**A**). Oligodendrocytes can engage in apoptosis via the activation of multiple signaling pathways in response to the FAS ligand (FASL), Tumor necrosis factor alpha (TNFα) or Interferon gamma (IFNγ) (**B**). Created with BioRender.com.

The pathogenesis of MS is believed to be autoimmune, involving local inflammation and lymphocytic destruction of oligodendrocytes, the myelinating cells of the CNS (Figure 1) [9]. Myelin is a protective sheath that covers the neuronal axons and is involved in the accelerated conduction of the action potential [10]. The autoimmune destruction of myelin impairs communication between neurons causing sensory, motor and cognitive dysfunctions [11]. The death of oligodendrocytes in MS occurs via multiple pathways (Figure 1). One of these is binding of the Fas ligand to its receptor on the surfaces of oligodendrocytes, leading to their caspase-8-mediated apoptosis. Indeed, immunohistochemistry displayed an increased expression of Fas on oligodendrocytes and Fas ligand on microglia cells in MS [12]. Another mechanism of oligodendrocyte death is through TNF-α mediated necrosis [13]. In accordance, a correlation was noted between the TNF-α concentration in the cerebrospinal fluid (CSF) and the severity of the disease presentation in MS [14]. The destruction of the oligodendrocytes can also be mediated by IFN-γ activated caspase-11, as it was demonstrated in caspase-11 knockout mice, where cells remained resistant to cell death upon IFN-γ treatment [15]. The putative role of pro-inflammatory cytokines and the extrinsic apoptotic pathway in the destruction of the oligodendrocytes fuels the idea of the autoimmune nature of MS.

## 3. Autoimmunity in Multiple Sclerosis

**Figure 2:**
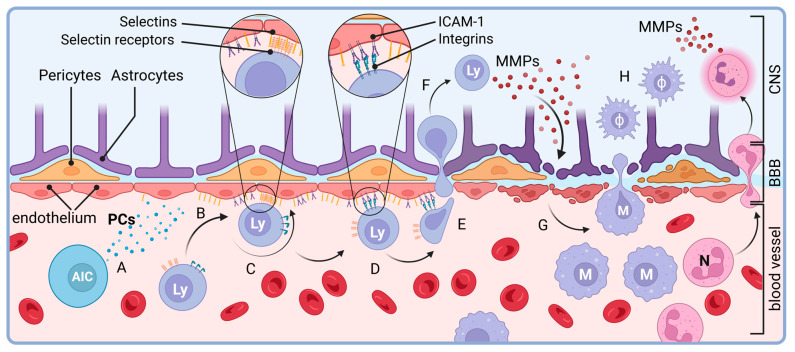
Pro-inflammatory macrophages invade the CNS in the initial phase of neuroinflammation in multiple sclerosis. Autoreactive immune cells (AIC)-released (A) pro-inflammatory cytokines (PC) induce the expression of adhesion molecules in endothelial cells that trigger recruitment (B) of lymphocytes (Ly), first, via weak Selectin (C) and, then, via firm, integrin-mediated (D) cell–cell interactions. The latter ones initiate diapedesis (E) of activated lymphocytes (F) into the interstitial space of CNS where secretion of matrix metalloproteinases (MMPs) damage *lamina basalis* of the blood–brain barrier (BBB). This leads to inflammatory injury of the cellular components of the blood–brain barrier including the endothelium, peri- and astrocytes alike. The damaged blood–brain barrier then recruits (G) monocytes (M) and neutrophil granulocytes (N). Following their immigration into the CNS, monocytes differentiate into pro-inflammatory macrophages (ϕ) and, with neutrophil granulocytes (N), initiate neuroinflammation (H). Created with BioRender.com.

Data indicate that upon the pathogenesis of MS, both innate and adaptive immune cells bypass the highly selective blood–brain barrier and become activated by myelin in a type IV hypersensitivity reaction [16]. It is believed that in MS patients, T lymphocytes are activated in the peripheral compartment and infiltrate the CNS leading to myelin and axonal damage [17]. The activation of autoreactive immune cells causes the secretion of cytokines, e.g., IL-1, IL-6 and TNF-α, compromising the blood–brain barrier (Figure 2) [18]. These pro-inflammatory cytokines upregulate the expression of adhesion molecules on vascular endothelial cells [19,20]. Lymphocytes bind to these adhesion molecules, migrate out of the vasculature and degrade the blood–brain barrier via matrix metalloproteases [21,22]. Early in the disease, macrophages dominate the infiltration followed by CD8^+^ and CD4^+^ T cells which, eventually, leads to general atrophy of the brain on imaging [23]. The majority of the CNS-infiltrating CD4^+^ T cells fall into the class of T_H_1 and T_H_17 cells, skewing the differentiation of CD4^+^ T cells away from the T_H_2 phenotype [24]. As the disease progresses, B- and plasma cells begin to accumulate in the connective tissue of CNS as well forming ectopic follicles [25]. Although it is unclear if the presence of follicles causes the severe disease or if it is rather the consequence of disease progression, the association between follicle formation and disease progression has been noted, [26,27]. Accordingly, immune cells are found to be autoreactive against myelin basic protein and proteolipid protein, components of the myelin sheets, and immunohistochemistry analyses identified C3d-, C9- and IgG-coated oligodendrocytes in the CNS of MS patients [28,29,30]. The role of autoreactive immune cells in the pathogenesis of the neuroinflammation in the CNS of MS patients has been supported by the findings that myelin basic protein-activated CD5^+^ T cells are able to provoke experimental allergic encephalomyelitis in healthy mice and that HLA-DR-positive macrophages are also present in the CSF of MS patients [31,32].

### 3.1. Macrophages

Besides monocytes recruited from the blood stream upon local inflammation, microglial cells, the tissue-resident macrophages of the CNS, are also believed to be key elements of MS pathogenesis. Tissue-resident macrophages are found locally in various tissues from birth where their tissue pools are maintained by in situ, macrophage colony-stimulating factor (M-CSF)/granulocyte macrophage colony-stimulating factor-dependent (GM-CSF) proliferation and play essential roles in immunosurveillance [33,34,35]. Embryologically, microglial cells are derived from primitive myeloid progenitors in the yolk sac, a distinct lineage compared to the hematopoietic stem cells, prior to the formation of the blood–brain barrier [36,37,38].

The putative role of microglial cells in the initial stages was established on the basis of the observation that the onset of the experimental autoimmune encephalomyelitis (EAE), the commonly used in vivo model of MS, was significantly delayed in a conditionally “paralyzed” microglial cell model [39]. It is believed that the activation of microglial cells leads to an oxidative burst causing free radical injury to the oligodendrocytes [40]. The progression from the initial stage, however, seems to be independent of microglial activation and rather dependent on the recruitment of monocytes from the blood [41]. This is mediated by the interaction between the monocyte chemoattractant protein-1 (MCP1) and the CC chemokine receptor 2 (CCR2) [42]. Indeed, macrophages are found in the CNS of MS patients and EAE models but in the absence of CCR2 on monocytes, progression of the EAE fails [43,44,45].

Macrophages that are usually present in acute and active inflammation sites are characterized by the surface markers MRP14 and 27E10. Monocytes migrate from blood vessels to sites of inflammation, followed by their differentiation into macrophages or dendritic cells (reviewed in [46]). Macrophages, then, develop either a pro-inflammatory M1 phenotype or an anti-inflammatory M2 phenotype [47,48]. Although in chronic MS, macrophages are typically positive for surface markers characteristic for matured species, in a relapsing model of EAE, an increase in circulating GM-CSF-activated CD11b^+^, CD62L^+^ and Ly6C(hi)^+^ pro-inflammatory monocytes was observed prior to relapse, suggesting that proinflammatory cells invade the CNS from extra-CNS reservoirs upon relapses [44,49]. The M1 phenotype secretes pro-inflammatory cytokines such as IFN-γ, TNF-alpha and IL-6 as well as reactive oxygen species leading to the death of myelinating cells in the CNS [50]. Since the macrophage-mediated disease progression in MS is, at least in part, GM-CSF-dependent that produced by a subset of T helper lymphocytes, T cells are believed to play a central role in MS pathogenesis [51].

### 3.2. T Lymphocytes

T lymphocytes are derived from hematopoietic stem cells in the bone marrow that travel to the thymus for maturation [52,53]. This process gives rise to a naïve T cell pool that does not react to autoantigens but is ready to recognize their cognate foreign antigens to mediate immune reaction (reviewed in [54]). The central role of autoreactive T cells in MS emerged from findings that susceptible animals can be prompted to develop EAE through the active induction or adoptive transfer of autoreactive myelin-specific lymphocytes [55,56,57]. These antigen-specific lymphocytes undergo colony expansion in the regional node before migrating to the central nervous system (CNS) and forming inflammatory centers, which result in symptoms analogous to those observed in MS. The concept of the central role of T cells in MS is further underpinned by the International Multiple Sclerosis Genetics Consortium showing that the strongest association with genetic risk factors is with the HLA-DRB*1501 and HLA-A*0301 MHC alleles, key mediators of T cell antigen recognition [58].

The two, apparently, most prominent fractions of T lymphocytes in MS lesions are the CD4^+^ T_H_1 and CD4^+^ T_H_17 populations expressing IFNγ and IL-17, respectively [59,60]. The latter one is believed to not only maintain T_H_17 cells in an autocrine manner but also to promote pro-inflammatory reactions within the CNS (reviewed in [61]). Besides IL-17, T_H_17 cells also secrete IL-21 and -22 which are thought to contribute to the destruction of the blood–brain barrier, supporting the invasion of the CNS by activated immune cells [62]

Upon thymic education, a subtype of T cells, termed regulatory T cells (T_reg_) and characterized by the CD4 and CD25 surface markers and the expression of the nuclear transcription factor FOXP3, also remain autoreactive, but that allows them to recognize and bind to autoantigens on the surface of other immune cells [63,64]. Indeed, T_reg_ cells help to maintain peripheral immunotolerance by inducing apoptosis in autoreactive T cells [65]. In the context of MS, depletion of T_reg_ cells suppresses the spontaneous recovery from EAE while an increase in T_reg_ cells is noted upon recovery from EAE [66]. In support of this concept, the induction of myelin oligodendrocyte glycoprotein-specific T_reg_ cells leads to the reduction of neuroinflammation in EAE models [67]. Data indicate that the recruitment of T_reg_ cells from the circulation upon remission in the EAE model is mediated via IL-4 [68]. The suppression of inflammation via T_reg_ cells is mediated via anti-inflammatory cytokines such as IL-10 [69]. IL-10 inhibits T cells by suppressing the co-stimulatory pathway for T cell activation via CD28 [70,71]. Indeed, EAE models with IL-10 knockout showed an increased release of pro-inflammatory cytokines [72]. In accordance, in MS patients, a functional defect in IL-10 secretion by T_reg_ cells was observed [73]. These observations fueled the agreement on the leading role of autoreactive T lymphocytes in MS.

### 3.3. B Cells

Although MS was considered as a primarily T cell dysregulation-mediated condition for years, recently this picture has been shifted toward a condition dictated by a disfunction between T and B lymphocytes. Indeed, the presence of oligoclonal immunoglobulins in the cerebrospinal fluid of MS patients and the positive effects of anti-CD20 antibodies in the clinical management of MS shed light on the importance of B cells in the pathogenesis of MS [74,75]. In secondary lymphoid organs, naïve B cells process antigens and load onto class II HLA complexes to present them for CD4^+^ Th lymphocytes, the key players in MS pathology [76]. Interestingly, the strongest genetic risk factor found in MS is the HLA-DRB*1501 allele that encodes for a class II MHC molecule [58]. In return, B cells respond to T_H_ cell-secreted IL-21 which trigger their switch to IgG^+^ species [77]. It is noteworthy that this is the same cytokine that is secreted by T_H_17 cells and is believed to play a role in the destruction of the blood–brain barrier in MS [78]. Thus, one can speculate whether the HLA-DRB*1501 allele and/or autoreactive T_H_17 cells compromise the reciprocal interaction between B and T cells in MS patients. Indeed, data indicate that, besides the autoreactive T lymphocytes, MS patients harbor defects in peripheral B cell tolerance checkpoints, leaving polyreactive B cells behind in the circulation [79]. Although details of the underlying mechanisms are still to be elucidated, dysregulated B cells eventually begin to express high levels of CXCR3, enabling them to cross the blood–brain barrier in MS patients [80]. Data indicate that in the CNS, CXCR3^+^ species differentiate into antibody-secreting cells that support the recruitment of further CD4^+^ T cells to the CNS [81].

### 3.4. Neutrophil Granulocytes

It is noteworthy, however, that the CD4^+^ T cell-driven EAE models that established the T cell-centered view of MS pathogenesis neither reproduce the full-blown human disease nor necessarily display the potential role of other immune cell subtypes in MS. Indeed, IL-17, for instance, is one of the cytokines that promotes the migration of IL-1β-producing neutrophil granulocytes to the CNS lymphatic system [82]. Although they were neglected initially in the context of this disease, neutrophil granulocytes are, now, believed to be potentially involved in the pathogenesis of MS as well. Indeed, neutrophil granulocyte products are detectable in the CSF during the onset of EAE, suggesting that the innate immune system also plays a role in the pathogenesis of MS [83]. This concept is further supported by findings that EAE animals show a delayed onset of EAE upon depletion of neutrophil granulocytes and that granulocyte macrophage-colony stimulating factor receptor knockout mice are resistant to EAE [84,85]. Moreover, the induction of genes encoding chemokines CXCL1 and CXCL2, which are known to be upregulated in the CNS during MS and are believed to orchestrate the chemotaxis of neutrophil granulocytes to the CSF and CNS in EAE, has been shown to be mediated by encephalitogenic CD4^+^ T_H_17 cells, the core players of MS pathogenesis [86]. In accordance, genetic ablation of the major receptor for CXCL1 and CXCL2 (CXCR2) abrogates EAE in mice, but the transplantation of CXCR2^+^ polymorphonuclear leukocytes into CXCR2^−/−^ animals is sufficient to restore susceptibility to EAE [86]. In humans, markers of the primed state of neutrophil granulocyte activity, like their elevated number, reduced apoptotic activity, higher expression of TLR-2, fMLP receptor, IL-8 receptor and CD43, enhanced degranulation and increased levels of neutrophil extracellular traps in serum, have also been documented, depicting a complex autoreactive status of the CNS in MS and fueling intensive investigations for factors involved in the dysregulation of immune cells upon Multiple Sclerosis [87].

## 4. Microbiome as a Regulatory Factor of the Immune System

MS epidemiology suggests that these include ethno-genetic factors since MS is more prevalent in Caucasians compared to individuals of African or Asian descent [88]. The highest prevalence is observed in Northern Europe, particularly in regions like the British Isles and Scandinavia, as well as areas initially settled by migrants from these regions, including North America, Australia, and New Zealand [89]. These epidemiologic data suggest a correlation between the prevalence of MS and the amount of winter sunlight, showing lower MS prevalence in regions with abundant sunlight [90]. This correlation, however, is not strong enough to consider sunlight exposure as the sole pathogenic factor of MS. Indeed, regions with very limited sunlight exposure, like the far north of Norway, show lower incident rates than that of Scotland or England and only half that of landlocked Oppland further south [91]. This peculiar pattern suggests the role of further ethno-genetic and/or geographic factors. Since latitude remains the most robustly associated factor of MS risk, inquiries into the role of photobiology linked vitamin D intake, particularly through multivitamin supplements, with a lower risk of MS [92,93,94]. A model that integrates vitamin D into genetic and environmental susceptibility to MS posits that vitamin D epigenetically modifies genes crucial in brain development, immune system function, axonal resilience, and immunological tolerance [90]. However, in a study assessing the effect of vitamin D supplementation on patients with relapsing–remitting multiple sclerosis (RR-MS), intriguing findings emerged, since vitamin D levels of RR-MS participants turned out to be very similar to that of the healthy participants both before and after 90 days of supplementation [95]. Instead, it was revealed that MS participants exhibited differential gut microbiota composition when compared to the healthy controls prior to supplementation of vitamin D. Specifically, they had lower levels of *Bacteroidaceae* and *Faecalibacterium*, and a higher abundance of *Ruminococcus*. It is particularly noteworthy that untreated MS subjects demonstrated a significant increase in the *Akkermansia*, *Faecalibacterium*, and *Coprococcus* genera following vitamin D supplementation [96]. These findings also raise the question of whether diet may influence MS incidence and progression, linking increased MS incidence to populations with high saturated fat intake and low vitamin D levels [90].

**Figure 3:**
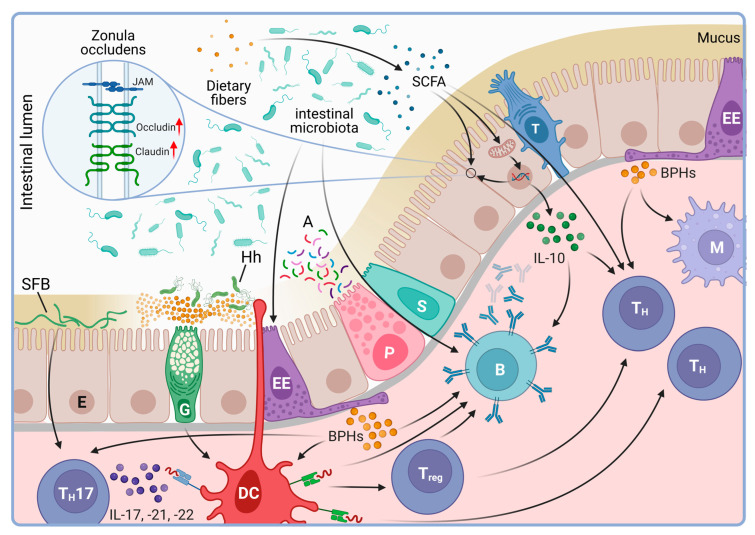
The gastrointestinal microbiome contributes to the functioning of the immune system. The gut microbiome and cellular elements of the intestinal epithelium have intricate interactions to maintain the barrier function of the gut lining. One of the critical components of this function is mediated by the tight junctions between enterocytes (E). Commensal germ metabolites, like short-chain fatty acids (SCFA), actively contribute to the upregulation of tight junction proteins. Parallel, enterocytes secrete cytokines that influence the immune cell population of the *lamina propria* including monocytes (M), antigen-producing cells (B) and helper (T_H_) and regulatory (T_reg_) T lymphocytes alike. Microbiota-derived antigens are also detected by enteroendocrine cells (EE), antigen presenting dendritic cells (DC) and enterocytes (E) by sampling the intestinal lumen directly. In return, enteroendocrine cells secrete bioactive peptide hormones (BPH), like serotonin, which, besides regulation of digestive functions of the enterocytes, affect B-, T_H_- and dendritic cell activities as well. The latter ones (DC), in contrast, can penetrate the intercellular space of the enterocyte lining and uptake microbial antigens directly from the mucus for presentation to immunocompetent cells of the epithelium. In the case of segmented filamentous bacteria (SFB), enterocytes can also mediate antigens to IL-17-producing T lymphocytes (T_H_17) contributing to the regulation of the functionally critical T_H_17 pool of the intestines. For this effect, SFB need to contact enterocytes by penetrating the lower layers of the mucus. This physical barrier primarily consists of mucin produced by Goblet cells (G) which also sample the gut microbiota for antigens, e.g., the *Helicobcter hepaticus* (Hh), and delivers them to elements of innate immune cells of the *lamina propria* like the dendritic species (DC). Goblet cells are, at least in certain conditions, under the control of Tuft cells (T) that, in response to luminal antigens, for instance upon helminth invasion, produce IL-25. IL-25 activates type 2 innate lymphoid and T helper cells that, in response to IL-25, begin to produce IL-13. IL-13, in return, facilitates the commitment of epithelial stem cells (S) toward the production of Goblet- (G) and Tuft cells (T) [108]. The mucus also contains antimicrobial peptides (A) secreted by various elements of the epithelium including Paneth cells (P), which usually present in close vicinity of epithelial stem cells (S) and that, at least in part, are under the indirect control of dendritic cells via cytokines including IL-22 [109]. Created with BioRender.com.

The potential involvement of dietary factors in MS pathogenesis naturally raises questions about the role of the microbiome (Figure 3). Indeed, emerging evidence suggests that the gastrointestinal microbiome plays an intricate role in shaping both innate and adaptive immunity, factors that are believed to be critical in the pathogenesis of MS. Studies on germ-free animals showed fundamental disturbances in the lymphoid tissue architecture and functions [97]. In the absence of the gut microbiota, IgA-mediated mucosal immunity is significantly reduced [98]. This is, at least in part, due to dendritic cells of the *lamina propria* that have been shown, sampling the mucosal layer of the intestinal lumen for microbial antigens both directly or through cellular elements of the gut epithelium like the Goblet cells, regulating immunoglobulin-producing B cells and T_reg_ lymphocytes alike [99,100,101]. *Helicobacter hepaticus* colonization in mice has also been shown to support T_reg_ responses through retinoic acid receptor-related orphan receptor γt-positive (RORγt^+^) species, a novel class of antigen-presenting cells [102,103]. The mucosal antigen presenting cells interact with the common members of the gut microbiome as well. The most immunodominant zwitterionic bacterial polysaccharide, polysaccharide A, has been shown to be internalized and presented on MHCII to activate CD4^+^ T cells [104]. Thus, one can speculate that the composition of the gut microbiome determines the development of the mucosal immune system. Indeed, studies involving microbiome isolates from healthy subjects and patients with inflammatory bowel disease (IBD) have revealed diverse dendritic cell cytokine responses, driven by differential engagement of host Toll-like receptors (TLRs) [105]. These characteristic cytokine profiles include interleukin 6 (IL-6), tumor necrosis factor α (TNFα), IL-10, IL-23 and IL-1β in response to *Bacteroidetes* and *Proteobacteria* in mouse myeloid cells in vitro [105]. Besides dendritic cells, segmented filamentous bacteria have also been shown to promote the development of antigen-specific T_H_17 cell responses underpinning the impact of microbiota on the regulation of the adaptive immune system [106,107].

Microbiome-produced metabolites, like short chain fatty acids, vitamin D3 or the trimethylamine N-oxide (TMAO), also seem to have significant effects on the immune system [110,111,112]. Of these, the SCFA butyrate is believed to have the highest biological importance. Acting as an energy source for the intestinal epithelium, butyrate contributes to the maintenance of the physiologic hypoxia of the intestinal epithelium, stabilizing the transcription factor hypoxia inducible factor 1 (HIF1) that, in turn, upregulates genes that maintain integrity of the epithelial barrier [113]. Butyrate also functions as a histone deacetylase inhibitor, and as such, it is believed to exert anti-inflammatory properties by modulating pro-inflammatory cytokine production via the inhibition of nuclear factor κB (NF-κB) [114,115]. Other metabolites, like indole, attenuate the intestinal immune system by fortifying tight junctions in the epithelium via the upregulation of junction proteins like Claudin and direct induction of anti-inflammatory cytokines like IL-10 [116].

TMAO, in contrast, directly induces pro-inflammatory mediators like TNFα, NLRP3 inflammasome, mitochondrial reactive oxygen species (ROS), and NF-κB, while reducing anti-inflammatory regulators such as IL-10 [117,118,119]. In accordance, TMAO triggers macrophage polarization toward the immunostimulatory M1 phenotype and enhances effector T cell functions, key factors of the pro-inflammatory state of the immune system [119]. These findings further support the idea of the potential relationship between the intestinal microbiota and the pathogenesis and progression of disorders associated with a dysregulation of the immune system like multiple sclerosis.

### 4.1. The Intestinal Microbiota

**Figure 4:**
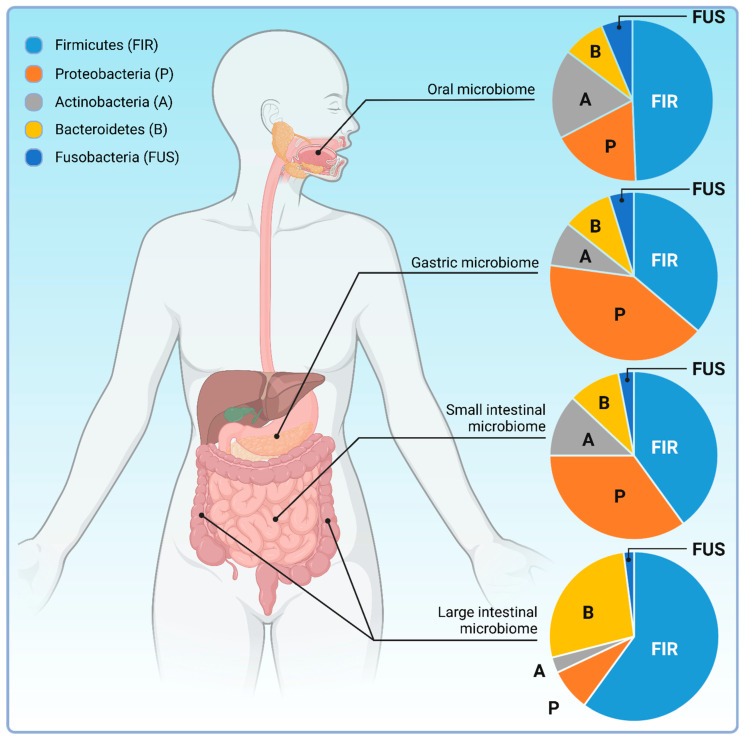
Spatially diverse composition of the gastrointestinal microbiome along the GI tract. Although predominant phyla are the same, proportional changes in microbiome diversity have been observed along the gastrointestinal tract of which the most characteristic is the reciprocal alteration in the presence of *Bacteroidetes/Firmucutes* and *Actino*-/*Proteobacteria* phyla in a cranio-caudal manner [129]. Created with BioRender.com.

The first recognition of the microbial presence in the GI tract of living animals dated back to the mid-1800s [120]. The microbial taxa present in the special environment of the human GI tract is a complex ecosystem that presents a collection of microbes and their genomes referred as the gastrointestinal microbiome [121]. Its characterization began in the early 20th century leading to the isolation of *Escherichia coli* by Alfred Nissle and the ongoing work depicts an intricate ecosystem that includes hundreds of microbial species [122]. Indeed, microbial communities have been identified along the entire GI tract, although microbiome composition of distinct sections of the GI tract is differential [123] (Figure 4). In general, the vast majority (>99%) of the identified species belong to five bacterial phyla including the *Firmicutes, Actinobacteria*, *Bacteroidetes*, *Proteobacteria* and *Fusobacteria* (Figure 4) [124,125]. The microbiota of the throat, that mostly constituted of the *Streptococcus*, *Prevotella*, *Actinomyces*, *Gemella*, *Rothia*, *Granulicatella*, *Haemophilus*, and *Veillonella* genera, shows the lowest phylotype diversity and inter-individual variety compared to the microbiota of the stomach or the lower GI tract [126]. Indeed, *Streptococcus*, *Actinomyces*, *Prevotella* and *Gemella* are the most abundant in the stomach [127]. In contrast, in fecal samples, representing the lower GI tract, genomic analyses revealed a minimum of 109 species from 8 phyla, 18 families, 23 classes, 38 orders and 59 genera [128]. According to current data, in fecal samples of healthy individuals, the phylum *Firmicutes* dominates (approx. 40%) [129]. The majority of the *Firmicutes* belong to the *Clostridia* class with frequent representation of the *Clostridium, Eubacterium* and *Ruminococcus* genera. The latter includes the most abundant species, the *Ruminococcus bicirculans*, that alone makes up approximately 2.5% of all species in the lower GI tract. The second most dominant phyla, both with an approximate 20% share in fecal samples, are the *Actinobacter,* that most abundantly comprises the species *Bifidobacterium longum* from the genus *Bifidobacterium,* and the *Bacteroidetes* mostly represented by the species *Bacteroides fragilis* from the class of *Bacteroidia*. At the species level, *Escherichia coli* (1.87% of total species) and the *Enterococcus faecium* (0.04% of total species) of the *Enterobacterales* and *Lactobacillales* orders, respectively, are the overrepresented in the lower GI tract [129].

### 4.2. Origin of the GI Microbiome

While distinct sections of the GI tract show differential microbiome composition, spatial distribution of the identified species is not the only diversity found in the GI tract microbiome. Genetic analyses revealed that the GI tract microbiome also shows significant age-related variability and data indicate that gestational age heavily affects human GI microbiota composition [130]. Fecal samples of very low birth-weight infants showed that, between 25 and 30 weeks of postmenstrual age, the lower GI microbiome is primarily dominated by *Staphylococci*. This population, then, is overtaken by a *Bifidobacterium*-dominated microbiota from postmenstrual week 30 onwards, accompanied by a peak in the abundance of *Enterococci* between postmenstrual weeks 30–35 and 45–50. In the case of term-born infants, the mouth flora was predominantly colonized by *Streptococci viridans* and *S. salivarius* by post-natal day 6 while the fecal flora showed the dominance of species from the classes of *Clostridia* and *Gammaproteobacteria* and the two most abundant species found were *Enterococcus faecalis* and *Escherichia coli* [130,131]. At the phyla level, it seems that delivery does not affect the colonization of the lower GI tract, since one month post-natal, fecal microbiota was found to be dominated by *Actinobacteria* independently of whether children were born via caesarean section (CS) or vaginal delivery (VD) [132]. Apparently, this changes at the later stages of the infant life since children’s fecal samples are dominated by *Bacteroidetes* and several genera of the *Clostridia* class, for example the *Ruminococci*, by the end of the first two years of age. At lower taxonomy levels, however, some degree of differential microbiome composition was observed much earlier post-natal. The *Enterococcus* genus, for instance, was found in significantly higher relative abundance in CS infants compared to their VD counterparts. In contrast, VD infants showed a significantly higher level of *Bacteroidetes* during the first 12 months post-natal. Parallel, CS delivery was found to be associated with a significantly lower overall microbiota diversity, even though the mothers of the CS and VD children did not show similar differences in their microbiota diversity. It is noteworthy, however, that the composition of the infant gut microbiota is affected by the feeding regime as well. Indeed, *Bacteroidetes* and *Clostridia* are more prevalent in fecal samples of weaned children than that of the breast-fed infants, highlighting the complexity of the determining factors of the gut microbiome [133,134].

### 4.3. Dietary Links

Similar to infants, dietary aspects affect the composition of the gut microbiome in adults as well. Animal studies revealed that the intake of non-digestible complex carbohydrates of plant origin, also known as dietary fibers or microbiome-accessible carbohydrates, is one of the determining factors of the gut microbiota diversity [135]. In humanized rodent models, a low microbiome-accessible carbohydrates diet primarily reduces the abundance of *Bacteroidetes*, the second most abundant phylum of the healthy human gut microbiome. The consumption of nutrients, e.g., in broccoli, in contrast, increases the abundance of *Bacteriodetes* [136]. The effect of nutrients on the diversity of the gut microbiome, however, is not uniform. Indeed, while walnut consumption increases the relative abundance of genera *Faecalibacterium, Clostridium, Roseburia,* and *Dialister* of the *Firmicutes* phylum, it decreases the abundance of *Bifidobacteria* of the phylum *Acinobacteria* [137]. An interesting aspect of the dietary effects on the gut microbiome is that, according to certain experimental findings, they can lead to the complete eradication of some species of the gut microbiota. A dietary supplement of microbiome-accessible carbohydrates to low level microbiome-accessible carbohydrates-fed animals alone did not reverse gut microbiome diversity if not combined with fecal microbiota transplants (FMT) [67]. Considering the significant role of nutrients in its composition, it is not surprising that the gut microbiota also shows seasonal changes. A long-term dietary survey analyzing the microbiota composition of human fecal samples showed that members of the phyla *Firmicutes* and *Actinobacteria* are more abundant in the samples collected over wintertime while species from the phylum *Bacteroidetes* are more abundant in samples taken in the summer months [70]. This seems to be in accordance with the findings that the abundance of *Bifidobacterium longum*, the most abundant species of *Bifidobacteriaceae*, positively correlates with the vegetable, protein and soluble fiber intake, or that *Akkermansia,* members of the phylum *Verrucomicrobia*, were shown to be positively associated with saturated fat intake and negatively correlated with the amount of total polyunsaturated fatty acids present in the diet [128]. Data also indicate that overall calorie intake is another important determinant of the gut microbiota diversity, showing a positive correlation between the body weight, the waist circumference and the abundance of certain species of the gut microbiota like *Bacteriodes ovatus* [138]. Indeed, fat-restricted or carbohydrate-restricted diets were found to increase the relative abundance of *Firmicutes* while decreasing the abundance of *Bacteriodetes*, underlining the importance of the dietary factors in the composition of the human gut microbiome [139].

### 4.4. Microbiome and Disease

Besides dietary factors, data suggest that the host’s genetic background also determines the gut microbiota composition. For instance, individuals carrying the HLA-DQ haplotype, a genetic condition that makes the individuals prone to Type I *Diabetes mellitus* (T1D), have a differential development course of their gut microbiome [140]. Indeed, the gut microbiome is dominated by members of the *Bacteroidetes* phylum in newborns during the first year post-natal, while, in children born with the HLA-DQ haplotype, the colonization of the gut by *Bacteroidetes* is delayed. In contrast, species representing the *Firmicutes* phylum peak early in the post-natal life of T1D-prone children, while they increase in abundance much later in non-HLA-DQ children. This early peak of colonization by *Firmicutes,* then, is accompanied by a depletion parallel with the increase in *Bacteroidetes* abundance, although, eventually, an overall reduction in the diversity of the gut microbiome of T1D-prone children by the age of year two was reported [140]. These data suggest that immune-related haplotypes interplay with the dynamics of the initial gut colonization, although it is not clear whether it is the gradually developing auto-immune environment that favors *Bacteroidetes* or the HLA-DQ haplotypes is the one that biases the gut microbiota toward *Bacteroidetes* which eventually induces the auto-immune condition leading to T1D.

The imbalance of *Bacteroidetes* and *Firmicutes* in the gut microbiome was documented in additional autoimmune pathologies too. In ulcerative colitis and Crohn’s disease patients, for instance, depletion of species from the *Firmicutes* phylum was reported to be more dominant over the depletion of *Bacteroidetes* [141]. In systemic lupus erythematosus, the prototype of the autoimmune diseases, similar dysbiosis was observed, indicating that the interplay of the gut microbiota and the immune homeostasis of the host environment is not restricted to the gastrointestinal tract [142]. Indeed, depletion of the *Firmicutes* phylum and increased abundance of species of the *Bacteroidetes* was also reported in patients suffering from rheumatoid arthritis [143]. In addition, imbalanced gut microbiota composition was reported in patients suffering from the major depressive disorder (MDD) [144]. Moreover, the depressive condition seemed to be transferrable via the gut microbiome, indicating the pathogenic importance of the disturbed gut microbiome in neuropsychological disorders. These observations further support the idea that the complex effects of the gastrointestinal microbiome affect tissues that were traditionally considered to be functionally independent of the GI tract.

Indeed, there is a growing body of evidence suggesting that an alteration in the GI tract microbiota diversity correlates with chronic human pathologies of neuronal tissue as well. In relation to Parkinson’s disease, for instance, the overall microbiota composition was found to be shifted with a depletion of butyrate-producing bacteria, and an overabundance of pro-inflammatory species like the *Collinsella*, *Desulfovibrio*, and *Oscillospiraceae* [145,146]. Even more interestingly, these alterations of the gut microbiome seem to be present in the long prodromal phase of Parkinson’s disease, suggesting a causality between the two processes [145]. In addition, a similar trend was found in REM sleep behavior disorder [145]. The predicted functional profile showed an overall increase in fatty acids’ fermentation to lactate and ethanol, and lower levels of deazapurine biosynthesis in both pathologies [145]. Reduced gut microbiome diversity has been reported in relation to Alzheimer’s disease as well. A recent meta-analysis of related studies showed an overall decrease in species richness in the Alzheimer’s disease gut microbiome, fueling the idea of the possible role of the GI tract microbiome in the pathogenesis of further neurodegenerative disorders [147].

## 5. The Intestinal Microbiome and Multiple Sclerosis

The regulatory role of the intestinal microbiome in shaping the immune system sheds light on its putative involvement in the onset and progression of multiple sclerosis. Accordingly, it turned out that the gut microbiome of MS patients showed dysbiosis mainly affecting genera of both *Firmicutes* and *Bacteroidetes* (Table 1 and Table 2) [148]. Interestingly, however, the trend of the observed changes seems to be independent of the phylogenetic classification. While *Bacteroidetes* genera *Pedobacteria* and *Flavobacterium* showed a higher abundance in patients with MS, other *Bacteroidetes* genera like the *Parabacteroides*, *Bacteroides*, and *Prevotella* were rather depleted. Moreover, it also seems that the diversity of the gut microbiome correlates with the disease course. Indeed, the small intestinal microbiota of patients suffering from RR-MS have an increase in the phylum *Firmicutes* and a decrease in the phylum *Bacteroidetes* during relapse compared to both healthy individuals and MS patients in remission [148,149,150].

**Figure 5:**
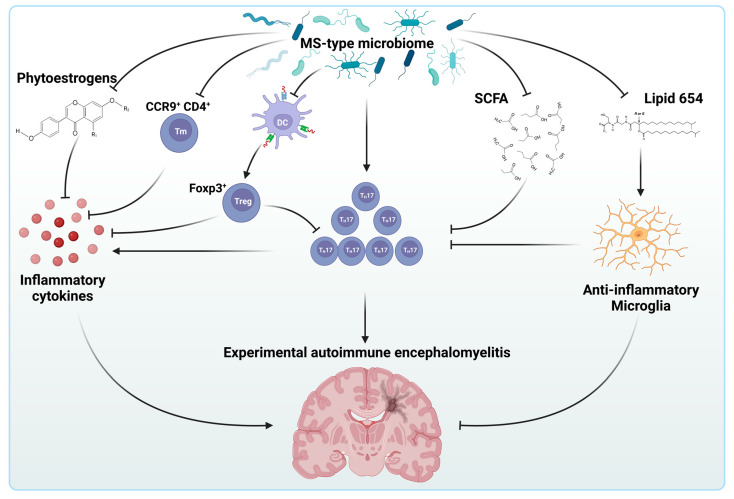
Dysbiosis of the gastrointestinal microbiome facilitates the proinflammatory state of multiple sclerosis. Alterations in the composition of the gastrointestinal microbiome influence the immunological status in multiple mechanisms in multiple sclerosis (MS). On one hand, MS-type microbiome directly facilitates differentiation and the expansion of T_H_17 lymphocytes (T_H_17) that play a central role in the maintenance of the inflammatory state of MS patients. Parallel, MS-type microbiome directly blocks the activity of anti-inflammatory species like the CCR9^+^ memory T cells (Tm) or tissue-resident dendritic cells (DC). Blockade of the latter results in the failure of differentiation of FOXP3^+^ regulatory T cells (T_reg_) which results in the accumulation of pro-inflammatory cytokines and cellular species alike. On the other hand, the MS-type intestinal microbiome exerts pro-inflammatory effects via various microbial metabolites by blocking the physiologic metabolisms of compounds like the phytoestrogens, short-chain fatty acids (SCFA) or microbial lipids (Lipid 654). Created with BioRender.com.

Although results on the gut microbiome diversity of MS patients are not entirely consistent, altering these ratios in animal models has been shown to influence disease phenotype. Susceptible animals develop EAE through the active induction or adoptive transfer of autoreactive myelin-specific lymphocytes [151]. These antigen-specific lymphocytes undergo colony expansion in the regional node before migrating to the CNS and forming inflammatory centers, which results in symptoms analogous to those observed in MS. The relapsing–remitting model of spontaneously developing experimental autoimmune EAE has been used to demonstrate the involvement of microbiota in disease development [152]. In mice, the transfer of intestinal microbiota from individuals with MS has been linked to the spontaneous development of EAE (Figure 5). Additionally, when mice are colonized with the gut microbes of MS patients, the resulting experimental disease is much more severe [153,154]. Interestingly, human alterations in gut microbiome in MS include a decrease in *Prevotella* and an increase in *Streptococcus mitis* (*S. mitis*) and *S. oralis*, all believed to have roles in shaping the immune system. Indeed, the *Prevotella* reduction is accompanied by the expansion of T_H_17 cells, while strains of the *S. mitis* group is known to positively affect the differentiation of T_H_17 cells, key players in cell-mediated tissue damage and autoimmunity in RR-MS patients [155,156]. Although the mechanistic relationship between these bacteria and the expansion of pro-inflammatory cell types like the T_H_17 cells are not fully understood, one may speculate that their immunological effects are mediated by the metabolites they produce.

Indeed, it is widely believed that the anti-inflammatory effects of bacteria like *Faecalibacterium* or *Coprococcusi* are primarily mediated by their butyrate production [157,158,159]. Similarly, *Prevotella* is a known propionate producer, that, along with acetate and butyrate, is a characteristic compound of the gut microbiome-produced short-chain fatty acids (SCFA) and is believed to be involved in the regulation of T_reg_ cells in peripheral compartments and the increase in the anti-inflammatory cytokine IL-10 [160,161,162,163]. To support this concept, dysbiosis of the gut microbiome in RR-MS patients has been observed to include the reduction of the *Clostridia* cluster XIV and IV accompanied by the reduction in the short-chain fatty acid production, also suggesting a widespread involvement of gut microbiota species in the unfolding of the pro-inflammatory state of MS via, at least in part, the reduction in short-chain fatty acid production [149].

Lipid 654, another bacterial metabolite typically produced by *Bacteroidetes* species that acts as a Toll-like receptor-2 ligand, was also found to be significantly lower in MS patients [164,165]. According to recent data, lipid 654 alters the microglial response to pro-inflammatory cytokines like IFNβ via shifting the microglial polarization to the rather anti-inflammatory M2 activation state [166]. Thus, the depletion of *Bacteroidetes* species in the MS gut microbiome might contribute to neuroinflammation via a reduction in lipid 654 production as well.

Besides the extensively studied *Firmicutes* and *Bacteroidetes* phyla, *Adlercreutzia* of the *Actinobacter* phylum has also been reported to be disturbed in MS patients’ microbiomes [148]. *Adlercreutzia* is known to play a role in influencing anti-inflammatory responses by way of its connection to phytoestrogen metabolism. Phytoestrogens are plant-derived molecules that possess a chemical structure and biological activity similar to estrogen. Legumes (especially soybeans), fruits, whole grains, and other vegetables are the primary sources of these compounds. Bacteria, such as *Adlercreutzia*, through β-glucosidase, are responsible for converting phytoestrogens into monomers. In patients with RR-MS, a reduction in *Adlercreutzia* leads to a decrease in the conversion capacity of phytoestrogens. Consequently, the decrease in this bacterium results in an increase in oxidative stress and inflammatory cytokines, such as the monocyte chemo-attractant protein-1 (MCP-1/CCL2) and IL-6, which are normally elevated in MS [167].

The compromised MS gut microbiome has been reported to have an impact on another chemokine–receptor interaction between the T-cell C-C chemokine receptor type 9 (CCR9) and its ligand CCL25 [168]. This interaction is important for CD4^+^ memory T cell homing to the gut and, thus, the immunity of the small intestinal epithelium [169]. CCR9^+^ CD4^+^ T cells belong to the intraepithelial cohort of T cells that has been found to be protective against EAE [170]. Disruption of the CCR9-CCL25 interaction by the MS gut microbiota results in a depleted population of CCR9^+^ CD4^+^ T cells, suggesting another potential mechanism involved in the exacerbation of neuroinflammation in SP-MS patients.

The putative link between the immunologic mechanisms and the gut microbiota in MS has been indirectly supported by findings in MS patients treated with glatiramer acetate (GA) [95]. Although GA was originally developed to provoke experimental autoimmune encephalitis as an animal model of human MS, it turned out to be acting as an immunomodulator and has been approved by the FDA as a first line drug to treat remitting MS [171]. GA is believed to reduce the secretion of pro-inflammatory cytokines (IL-2, IL-12, IFNγ, TNF) released by T_H_1 cells, activate T_H_2 suppressor cells to express anti-inflammatory cytokines (IL-4, IL-5, IL-13, IL-10, TGF-β) in the CNS and increase the enrichments of T_regs_ [172,173]. GA, however, has also been shown to modulate the composition of the gut microbiota in MS patients elevating the proportion of *Janthinobacterium* while decreasing the presence of *Eubacterium* and *Ruminococcus*, suggesting the a putative bidirectional interplay between the gut microbiota and the immune mechanisms involved in MS [95].

These observations inevitably raise the question of the causality in the interplay between the intestinal microbiome and the immune system in MS. The two main hypotheses of the pathogeneses of MS allows differential interpretation of the role of the intestinal microbiome in the development of the disease. The inside-out concept posits that MS is initiated by a primary lesion in the CNS, potentially triggered by factors like infection or primary neurodegeneration. This event leads to the release of self-antigens, provoking a response from autoreactive T and/or B cells. In this concept, one could speculate that the differential composition of the gut microbiome observed in MS patients is a consequence of a preexisting pro-inflammatory state of the immune system.

Conversely, the extrinsic or outside-in concept suggests that peripheral stimuli like microbial antigens resembling CNS molecular signatures, bystander activation of immunocompetent cells, novel autoantigen presentation, or recognition of secluded CNS antigens activate escaped autoreactive T cells, eventually leading to a neuroinflammatory state [174]. In this concept, disturbances in the gut microbiome might play the role of the initial antigen stimulus. This idea is, apparently, strongly supported by animal studies in EAE models. Indeed, the administration of differential combinations of antibiotics inhibits the development of EAE in susceptible animals. This disruption is linked to an increase in FOXP3^+^ regulatory T cells in the mesenteric and cervical lymph nodes which appear to be influenced by an elevated presence of the co-localized CD11c^high^CD103^+^ dendritic cells. These specific DCs promote the transformation of naïve CD4^+^ T cells into FOXP3^+^ T_reg_ cells [175]. Parallel, an increased presence of IL-10-producing CD5^+^ B cells has also been observed in cervical lymph nodes. When splenic CD5^+^ B cells from antibiotic-treated mice were transferred to naïve recipient mice, and the recipients were immunized with MOG35–55 one day post-transplantation, a significant reduction in EAE disease score was noted. In addition, this reduction seems to be associated with a shift from a T_H_1/T_H_17 cytokine profile to a T_H_2 cytokine profile [176]. To further support the role of the gut microbiome in the extrinsic model of MS, the transplantation of the intestinal microbiota of MS patients into susceptible mice has been observed to be associated with the spontaneous onset of EAE. Moreover, when mice are colonized with microbes sourced from the gut of MS patients, the resultant experimental disease manifests with increased severity, suggesting a causative role of the microbiome in the development of MS in susceptible individuals [153,154].

Considering its complexity, one can speculate that the intestinal microbiome can provide a generous source of non-self biocompounds that could serve by triggering antigens for autoreactive species. Research identifying the guanosine diphosphate-L-fucose synthase as an autoantigen for CD4^+^ autoreactive T cells supports this idea [177]. This enzyme is involved in the nucleotide–sugar biosynthesis and catalyzes the conversion of GDP-4-dehydro-6-deoxy-D-mannose to GDP-fucose which serves as substrate for fucosyltransferases to the mediated fusosylation of oligosaccharides, glycoproteins, and glycolipids [178]. Although, in mammals, fucosylation plays a critically important role in a number of physiologic processes, in prokaryotes fucosylation is less common. Still, it has been demonstrated that certain bacteria of the gastrointestinal tract, like oral commensals that are associated with infected endocarditis, such as *Porphyromonas gingivalis, Actinobacillus actinomycetemcomitans* and *Eikenella corrodens* or the gastric *Helicobacter pylori*, exploit fucosylated structures on bacterial lipopolysaccharides (LPS) [179]. Indeed, in the latter, for instance, molecular mimicry through fucosylated LPS enhances *H. pylori* adhesion to the gastric epithelium and modulates hosts’ immune responses to aid adaptation effectively to its niche and maintain a persistent infection [180]. Interestingly, the fucosylated polysaccharide chains of *H. pylori* LPS induce the production of auto-antibodies, causing antigastric autoreactivity and, consequently, tissue damage in the gastric mucosa [181].

Thus, one can speculate that the CD4^+^ T lymphocyte-mediated immune reaction against the guanosine diphosphate-L-fucose synthase is, primarily, a protective measure against potentially pathogenic invading microbes in the gastrointestinal tract that, eventually, turns into autoreactivity either due to partial molecular homology between the microbial and host guanosine diphosphate-L-fucose synthase or the genetic constellation of the host. The latter concept seems to be supported by the findings that there are significant associations between the reactivity to GDP-L-fucose synthase peptides along with reactivity against an immunodominant myelin basic protein peptide and the HLA-DRB3*02:02 haplotype, one of the MS-associated genetic constellation found by the International Multiple Sclerosis Genetics Consortium [58].

Interestingly, fucosylated glycans are highly expressed in brain tissue and human CNS myelin compared with other tissues and support recognition by microglia and dendritic cells [182,183]. Thus, one can speculate that the absence of this tolerogenic signal upon reduced fucosylation due to the potentially intestinal microbiome-mediated occurrence of GDP-L-fucose synthase-reactive CD4^+^ T cells contributes to the neuroinflammatory state of the CNS of MS patients.

## 6. Conclusions

The prevalence of multiple sclerosis has significantly increased over the past three decades, putting a huge economic burden on healthcare systems worldwide [184,185]. The increase in prevalence is attributed to increased incidence, improvement in patient care leading to an increased survival of patients and better diagnostic tools [186,187].

Despite the great efforts made in the research of the disease, details of the pathogenesis remain elusive. Although there is a wide consensus on the autoimmune nature of MS, little is known of the stimuli that trigger the disease or the conditions that make individuals prone to MS. Indeed, although the correlation between certain genotypes or pathogens and the risk of MS has been postulated, the nature of the role of these putative risk factors is still not clear. Advances in the identification of key players in the symptomatic phases of MS, however, could take us closer to answering this question.

T_H_17 cells have been identified as one of the key players in the pathogenesis of MS. Their hallmark cytokine product, IL-17, however, was found to be protective in both *K. pneummoniae* infections and in asthma models, where it reduced the eosinophilia and bronchial hyper-reactivity alike, suggesting a *bona fide* role of T_H_17 cells in these immune situations [188,189]. Moreover, IL-17 was shown to control the integrity of tight junctions in the intestinal epithelium by influencing the subcellular localization of occludin, a critical component of *zonula occludens* [190]. These interactions immediately provoke the question of whether similar microbial-T_H_17 links give rise to the myelin-reactive populations of T_H_17 cells via, for instance, molecular mimicry and, if so, whether analogous interplays exist between members of the gastrointestinal microbiome and T_H_17 lymphocytes.

Indeed, data on the gut microbiome diversity in monozygotic twins discordant to MS, for instance, suggest that the proposed genetic background is not sufficient for the development of the disease, modulating the picture on the importance of genetic risk factors in MS further [153]. Moreover, the microbiomes of MS-twins can provoke autoimmunity accompanied by paralyzed production of the anti-inflammatory IL-10 following transplantation in susceptible animal models, fueling the idea of the pathogenic role of the gut microbiome mediated via immunological elements in MS.

High individual diversity of gut microbiomes may prove the identification of pathogenic species to be particularly difficult, suggesting that research on the role of the human microbiome in diseases like MS should, perhaps, be focused on the characteristics of the protective microbiome composition. Observations that increasing the abundance of *Adlercreutzia* in the gut microbiome of RR-MS patients leads to a decrease in the inflammatory state and consequently delays the progression of MS apparently support this idea, and raise the question of whether therapeutic alteration of the gut microbiome could be useful in the treatment of MS patients.

Indeed, fecal microbiota transplantation (FMT) has been successfully used in diverse conditions including severe dysbiosis due to refractory and recurrent *C. difficile* infections [191]. Although concerns of FMT include the invasiveness of the intervention, the modest but significant risk of infection transmission, thorough donor screening or transplantation of bacteria-free filtrates have already been demonstrated as measures to minimize the risk factors of FMT [192,193]. Following this logic, the use of FMT in MS has been recently tested in a small scale pilot study demonstrating its safety and tolerability, suggesting a new avenue in the treatment of multiple sclerosis [194].

Due to its apparently overwhelming complexity, one can speculate that a great proportion of the mechanism underlying the pathology of MS is highly individual. If that complexity exists, it calls for further investigations to identify mechanisms common in MS patients that could serve as potential targets for developing successful treatment regimes. It is believed that Hippocrates, the forerunner of modern evidence-based medicine, said that “People think that epilepsy is divine simply because they don’t have any idea what causes epilepsy. But I believe that, someday, we will understand what causes epilepsy, and at that moment, we will cease to believe that it’s divine. And so, it is with everything in the universe”. Indeed, life sciences have been on a great journey since the era of Hippocrates and solved several mysteries woven together with the human body, achievements Hippocrates could not even have dreamed of. Still, we, perhaps, did not pay enough attention to his observation when he said, “All disease starts in the gut”. In the light of advances in the field of intestinal physiology in recent years, it seems that another Hippocrates’ quote, “Let food be thy medicine and medicine be thy food”, perhaps, has never been timelier.

## Figures and Tables

**Table 1:** Genera of gut microbiota with increased abundance in multiple sclerosis.

*Phylum*	*Genus*
*Bacteroidetes*	*Pedobacteria* *Flavobacterium*
*Firmicutes*	*Dorea* *Balutia* *Streptococcus*
*Proteobacteria*	*Mycoplana* *Acinetobacter* *Pseudomonas*
*Actinobacteria*	*Eggerthella*
*Verrucomicrobia*	*Akkermansia*

**Table 2:** Genera of gut microbiota with decreased abundance in multiple sclerosis.

*Phylum*	*Genus*
*Bacteroidetes*	*Bacteroides* *Prevotella* *Parabacteroides*
*Firmicutes*	*Coprobacillus* *Lactobacillus* *Clostridium* *Anaerostipes* *Faecalibacterium*
*Proteobacteria*	*Haemophylus* *Sutterella*
*Actinobacteria*	*Adlercreutzia* *Collinsella*

## Data Availability

Data sharing is not applicable to this article.

## References

[1] Wylezinski L.S., Gray J.D., Polk J.B., Harmata A.J., Spurlock C.F. (2019). Illuminating an Invisible Epidemic: A Systemic Review of the Clinical and Economic Benefits of Early Diagnosis and Treatment in Inflammatory Disease and Related Syndromes. J. Clin. Med..

[2] GBD 2015 Neurological Disorders Collaborator Group (2017). Global, regional, and national burden of neurological disorders during 1990–2015: A systematic analysis for the Global Burden of Disease Study 2015. Lancet Neurol..

[3] Sawcer S., Jones H.B., Feakes R., Gray J., Smaldon N., Chataway J., Robertson N., Clayton D., Goodfellow P.N., Compston A. (1996). A genome screen in multiple sclerosis reveals susceptibility loci on chromosome 6p21 and 17q22. Nat. Genet..

[4] Ben-Zacharia A.B. (2011). Therapeutics for multiple sclerosis symptoms. Mt. Sinai J. Med..

[5] Betts C.D., D’Mellow M.T., Fowler C.J. (1993). Urinary symptoms and the neurological features of bladder dysfunction in multiple sclerosis. J. Neurol. Neurosurg. Psychiatry.

[6] Patten S.B., Beck C.A., Williams J.V., Barbui C., Metz L.M. (2003). Major depression in multiple sclerosis: A population-based perspective. Neurology.

[7] Lublin F.D., Reingold S.C., Cohen J.A., Cutter G.R., Sorensen P.S., Thompson A.J., Wolinsky J.S., Balcer L.J., Banwell B., Barkhof F. (2014). Defining the clinical course of multiple sclerosis: The 2013 revisions. Neurology.

[8] Gohil K. (2015). Multiple Sclerosis: Progress, but No Cure. Pharm. Ther..

[9] de Waegh S., Brady S.T. (1990). Altered slow axonal transport and regeneration in a myelin-deficient mutant mouse: The trembler as an in vivo model for Schwann cell-axon interactions. J. Neurosci..

[10] McDonald W.I., Sears T.A. (1969). Effect of demyelination on conduction in the central nervous system. Nature.

[11] Anlar O., Tombul T., Kisli M. (2003). Peripheral sensory and motor abnormalities in patients with multiple sclerosis. Electromyogr. Clin. Neurophysiol..

[12] D’Souza S.D., Bonetti B., Balasingam V., Cashman N.R., Barker P.A., Troutt A.B., Raine C.S., Antel J.P. (1996). Multiple sclerosis: Fas signaling in oligodendrocyte cell death. J. Exp. Med..

[13] Selmaj K.W., Raine C.S. (1988). Tumor necrosis factor mediates myelin and oligodendrocyte damage in vitro. Ann. Neurol..

[14] Sharief M.K., Hentges R. (1991). Association between tumor necrosis factor-alpha and disease progression in patients with multiple sclerosis. N. Engl. J. Med..

[15] Hisahara S., Yuan J., Momoi T., Okano H., Miura M. (2001). Caspase-11 mediates oligodendrocyte cell death and pathogenesis of autoimmune-mediated demyelination. J. Exp. Med..

[16] Isabwe G.A.C., Garcia Neuer M., de Las Vecillas Sanchez L., Lynch D.M., Marquis K., Castells M. (2018). Hypersensitivity reactions to therapeutic monoclonal antibodies: Phenotypes and endotypes. J. Allergy Clin. Immunol..

[17] Engelhardt B. (2010). T cell migration into the central nervous system during health and disease: Different molecular keys allow access to different central nervous system compartments. Clin. Exp. Neuroimmunol..

[18] Correale J., Gilmore W., McMillan M., Li S., McCarthy K., Le T., Weiner L.P. (1995). Patterns of cytokine secretion by autoreactive proteolipid protein-specific T cell clones during the course of multiple sclerosis. J. Immunol..

[19] Zhang J., Alcaide P., Liu L., Sun J., He A., Luscinskas F.W., Shi G.P. (2011). Regulation of endothelial cell adhesion molecule expression by mast cells, macrophages, and neutrophils. PLoS ONE.

[20] Kawachi S., Jennings S., Panes J., Cockrell A., Laroux F.S., Gray L., Perry M., van der Heyde H., Balish E., Granger D.N. (2000). Cytokine and endothelial cell adhesion molecule expression in interleukin-10-deficient mice. Am. J. Physiol. Gastrointest. Liver Physiol..

[21] Sellebjerg F., Sorensen T.L. (2003). Chemokines and matrix metalloproteinase-9 in leukocyte recruitment to the central nervous system. Brain Res. Bull..

[22] Bar-Or A., Nuttall R.K., Duddy M., Alter A., Kim H.J., Ifergan I., Pennington C.J., Bourgoin P., Edwards D.R., Yong V.W. (2003). Analyses of all matrix metalloproteinase members in leukocytes emphasize monocytes as major inflammatory mediators in multiple sclerosis. Brain.

[23] Chard D.T., Griffin C.M., Parker G.J., Kapoor R., Thompson A.J., Miller D.H. (2002). Brain atrophy in clinically early relapsing-remitting multiple sclerosis. Brain.

[24] Tzartos J.S., Friese M.A., Craner M.J., Palace J., Newcombe J., Esiri M.M., Fugger L. (2008). Interleukin-17 production in central nervous system-infiltrating T cells and glial cells is associated with active disease in multiple sclerosis. Am. J. Pathol..

[25] Frischer J.M., Bramow S., Dal-Bianco A., Lucchinetti C.F., Rauschka H., Schmidbauer M., Laursen H., Sorensen P.S., Lassmann H. (2009). The relation between inflammation and neurodegeneration in multiple sclerosis brains. Brain.

[26] Magliozzi R., Howell O., Vora A., Serafini B., Nicholas R., Puopolo M., Reynolds R., Aloisi F. (2007). Meningeal B-cell follicles in secondary progressive multiple sclerosis associate with early onset of disease and severe cortical pathology. Brain.

[27] Howell O.W., Reeves C.A., Nicholas R., Carassiti D., Radotra B., Gentleman S.M., Serafini B., Aloisi F., Roncaroli F., Magliozzi R. (2011). Meningeal inflammation is widespread and linked to cortical pathology in multiple sclerosis. Brain.

[28] Yandamuri S.S., Filipek B., Obaid A.H., Lele N., Thurman J.M., Makhani N., Nowak R.J., Guo Y., Lucchinetti C.F., Flanagan E.P. (2023). MOGAD patient autoantibodies induce complement, phagocytosis, and cellular cytotoxicity. JCI Insight.

[29] Steinman L. (1999). Absence of “original antigenic sin” in autoimmunity provides an unforeseen platform for immune therapy. J. Exp. Med..

[30] Wucherpfennig K.W., Zhang J., Witek C., Matsui M., Modabber Y., Ota K., Hafler D.A. (1994). Clonal expansion and persistence of human T cells specific for an immunodominant myelin basic protein peptide. J. Immunol..

[31] Pettinelli C.B., McFarlin D.E. (1981). Adoptive transfer of experimental allergic encephalomyelitis in SJL/J mice after in vitro activation of lymph node cells by myelin basic protein: Requirement for Lyt 1^+^ 2^−^ T lymphocytes. J. Immunol..

[32] Gay D., Esiri M. (1991). Blood-brain barrier damage in acute multiple sclerosis plaques. An immunocytological study. Brain.

[33] Nimmerjahn A., Kirchhoff F., Helmchen F. (2005). Resting microglial cells are highly dynamic surveillants of brain parenchyma in vivo. Science.

[34] Hashimoto D., Chow A., Noizat C., Teo P., Beasley M.B., Leboeuf M., Becker C.D., See P., Price J., Lucas D. (2013). Tissue-resident macrophages self-maintain locally throughout adult life with minimal contribution from circulating monocytes. Immunity.

[35] Lavin Y., Mortha A., Rahman A., Merad M. (2015). Regulation of macrophage development and function in peripheral tissues. Nat. Rev. Immunol..

[36] Ginhoux F., Greter M., Leboeuf M., Nandi S., See P., Gokhan S., Mehler M.F., Conway S.J., Ng L.G., Stanley E.R. (2010). Fate mapping analysis reveals that adult microglia derive from primitive macrophages. Science.

[37] Schulz C., Gomez Perdiguero E., Chorro L., Szabo-Rogers H., Cagnard N., Kierdorf K., Prinz M., Wu B., Jacobsen S.E., Pollard J.W. (2012). A lineage of myeloid cells independent of Myb and hematopoietic stem cells. Science.

[38] Ajami B., Bennett J.L., Krieger C., Tetzlaff W., Rossi F.M. (2007). Local self-renewal can sustain CNS microglia maintenance and function throughout adult life. Nat. Neurosci..

[39] Heppner F.L., Greter M., Marino D., Falsig J., Raivich G., Hovelmeyer N., Waisman A., Rulicke T., Prinz M., Priller J. (2005). Experimental autoimmune encephalomyelitis repressed by microglial paralysis. Nat. Med..

[40] Fischer M.T., Sharma R., Lim J.L., Haider L., Frischer J.M., Drexhage J., Mahad D., Bradl M., van Horssen J., Lassmann H. (2012). NADPH oxidase expression in active multiple sclerosis lesions in relation to oxidative tissue damage and mitochondrial injury. Brain.

[41] Ajami B., Bennett J.L., Krieger C., McNagny K.M., Rossi F.M. (2011). Infiltrating monocytes trigger EAE progression, but do not contribute to the resident microglia pool. Nat. Neurosci..

[42] Izikson L., Klein R.S., Charo I.F., Weiner H.L., Luster A.D. (2000). Resistance to experimental autoimmune encephalomyelitis in mice lacking the CC chemokine receptor (CCR)2. J. Exp. Med..

[43] Polman C.H., Dijkstra C.D., Sminia T., Koetsier J.C. (1986). Immunohistological analysis of macrophages in the central nervous system of Lewis rats with acute experimental allergic encephalomyelitis. J. Neuroimmunol..

[44] Bruck W., Sommermeier N., Bergmann M., Zettl U., Goebel H.H., Kretzschmar H.A., Lassmann H. (1996). Macrophages in multiple sclerosis. Immunobiology.

[45] Fife B.T., Huffnagle G.B., Kuziel W.A., Karpus W.J. (2000). CC chemokine receptor 2 is critical for induction of experimental autoimmune encephalomyelitis. J. Exp. Med..

[46] Ginhoux F., Guilliams M. (2016). Tissue-Resident Macrophage Ontogeny and Homeostasis. Immunity.

[47] Mikita J., Dubourdieu-Cassagno N., Deloire M.S., Vekris A., Biran M., Raffard G., Brochet B., Canron M.H., Franconi J.M., Boiziau C. (2011). Altered M1/M2 activation patterns of monocytes in severe relapsing experimental rat model of multiple sclerosis. Amelioration of clinical status by M2 activated monocyte administration. Mult. Scler..

[48] Liu C., Li Y., Yu J., Feng L., Hou S., Liu Y., Guo M., Xie Y., Meng J., Zhang H. (2013). Targeting the shift from M1 to M2 macrophages in experimental autoimmune encephalomyelitis mice treated with fasudil. PLoS ONE.

[49] King I.L., Dickendesher T.L., Segal B.M. (2009). Circulating Ly-6C+ myeloid precursors migrate to the CNS and play a pathogenic role during autoimmune demyelinating disease. Blood.

[50] Kigerl K.A., Gensel J.C., Ankeny D.P., Alexander J.K., Donnelly D.J., Popovich P.G. (2009). Identification of two distinct macrophage subsets with divergent effects causing either neurotoxicity or regeneration in the injured mouse spinal cord. J. Neurosci..

[51] Codarri L., Gyulveszi G., Tosevski V., Hesske L., Fontana A., Magnenat L., Suter T., Becher B. (2011). RORgammat drives production of the cytokine GM-CSF in helper T cells, which is essential for the effector phase of autoimmune neuroinflammation. Nat. Immunol..

[52] Akashi K., Kondo M., Weissman I.L. (1998). Role of interleukin-7 in T-cell development from hematopoietic stem cells. Immunol. Rev..

[53] Egerton M., Scollay R., Shortman K. (1990). Kinetics of mature T-cell development in the thymus. Proc. Natl. Acad. Sci. USA.

[54] Stritesky G.L., Jameson S.C., Hogquist K.A. (2012). Selection of self-reactive T cells in the thymus. Annu. Rev. Immunol..

[55] Kabat E.A., Wolf A., Bezer A.E. (1947). The Rapid Production of Acute Disseminated Encephalomyelitis in Rhesus Monkeys by Injection of Heterologous and Homologous Brain Tissue with Adjuvants. J. Exp. Med..

[56] Paterson P.Y. (1960). Transfer of allergic encephalomyelitis in rats by means of lymph node cells. J. Exp. Med..

[57] Arnason B.G., Jankovic B.D., Waksman B.H., Wennersten C. (1962). Role of the thymus in immune reactions in rats. II. Suppressive effect of thymectomy at birth on reactions of delayed (cellular) hypersensitivity and the circulating small lymphocyte. J. Exp. Med..

[58] International Multiple Sclerosis Genetics C., Wellcome Trust Case Control C., Sawcer S., Hellenthal G., Pirinen M., Spencer C.C., Patsopoulos N.A., Moutsianas L., Dilthey A., Su Z. (2011). Genetic risk and a primary role for cell-mediated immune mechanisms in multiple sclerosis. Nature.

[59] Brucklacher-Waldert V., Stuerner K., Kolster M., Wolthausen J., Tolosa E. (2009). Phenotypical and functional characterization of T helper 17 cells in multiple sclerosis. Brain.

[60] Voskuhl R.R., Martin R., Bergman C., Dalal M., Ruddle N.H., McFarland H.F. (1993). T helper 1 (Th1) functional phenotype of human myelin basic protein-specific T lymphocytes. Autoimmunity.

[61] Milovanovic J., Arsenijevic A., Stojanovic B., Kanjevac T., Arsenijevic D., Radosavljevic G., Milovanovic M., Arsenijevic N. (2020). Interleukin-17 in Chronic Inflammatory Neurological Diseases. Front. Immunol..

[62] Kebir H., Kreymborg K., Ifergan I., Dodelet-Devillers A., Cayrol R., Bernard M., Giuliani F., Arbour N., Becher B., Prat A. (2007). Human TH17 lymphocytes promote blood-brain barrier disruption and central nervous system inflammation. Nat. Med..

[63] Dieckmann D., Plottner H., Berchtold S., Berger T., Schuler G. (2001). Ex vivo isolation and characterization of CD4^+^CD25^+^ T cells with regulatory properties from human blood. J. Exp. Med..

[64] Lio C.W., Hsieh C.S. (2008). A two-step process for thymic regulatory T cell development. Immunity.

[65] Kohm A.P., Carpentier P.A., Anger H.A., Miller S.D. (2002). Cutting edge: CD4^+^CD25^+^ regulatory T cells suppress antigen-specific autoreactive immune responses and central nervous system inflammation during active experimental autoimmune encephalomyelitis. J. Immunol..

[66] McGeachy M.J., Stephens L.A., Anderton S.M. (2005). Natural recovery and protection from autoimmune encephalomyelitis: Contribution of CD4^+^CD25^+^ regulatory cells within the central nervous system. J. Immunol..

[67] Chung D.T., Korn T., Richard J., Ruzek M., Kohm A.P., Miller S., Nahill S., Oukka M. (2007). Anti-thymocyte globulin (ATG) prevents autoimmune encephalomyelitis by expanding myelin antigen-specific FOXP3+ regulatory T cells. Int. Immunol..

[68] Butti E., Bergami A., Recchia A., Brambilla E., Del Carro U., Amadio S., Cattalini A., Esposito M., Stornaiuolo A., Comi G. (2008). IL4 gene delivery to the CNS recruits regulatory T cells and induces clinical recovery in mouse models of multiple sclerosis. Gene Ther..

[69] Asseman C., Mauze S., Leach M.W., Coffman R.L., Powrie F. (1999). An essential role for interleukin 10 in the function of regulatory T cells that inhibit intestinal inflammation. J. Exp. Med..

[70] Akdis C.A., Joss A., Akdis M., Faith A., Blaser K. (2000). A molecular basis for T cell suppression by IL-10: CD28-associated IL-10 receptor inhibits CD28 tyrosine phosphorylation and phosphatidylinositol 3-kinase binding. FASEB J..

[71] Joss A., Akdis M., Faith A., Blaser K., Akdis C.A. (2000). IL-10 directly acts on T cells by specifically altering the CD28 co-stimulation pathway. Eur. J. Immunol..

[72] Bettelli E., Das M.P., Howard E.D., Weiner H.L., Sobel R.A., Kuchroo V.K. (1998). IL-10 is critical in the regulation of autoimmune encephalomyelitis as demonstrated by studies of IL-10- and IL-4-deficient and transgenic mice. J. Immunol..

[73] Astier A.L., Hafler D.A. (2007). Abnormal Tr1 differentiation in multiple sclerosis. J. Neuroimmunol..

[74] Annunziata P., Giorgio A., De Santi L., Zipoli V., Portaccio E., Amato M.P., Clerici R., Scarpini E., Moscato G., Iudice A. (2006). Absence of cerebrospinal fluid oligoclonal bands is associated with delayed disability progression in relapsing-remitting MS patients treated with interferon-beta. J. Neurol. Sci..

[75] de Seze J., Maillart E., Gueguen A., Laplaud D.A., Michel L., Thouvenot E., Zephir H., Zimmer L., Biotti D., Liblau R. (2023). Anti-CD20 therapies in multiple sclerosis: From pathology to the clinic. Front. Immunol..

[76] Yuseff M.I., Lennon-Dumenil A.M. (2015). B Cells use Conserved Polarity Cues to Regulate Their Antigen Processing and Presentation Functions. Front. Immunol..

[77] Mitsdoerffer M., Lee Y., Jager A., Kim H.J., Korn T., Kolls J.K., Cantor H., Bettelli E., Kuchroo V.K. (2010). Proinflammatory T helper type 17 cells are effective B-cell helpers. Proc. Natl. Acad. Sci. USA.

[78] Dvorscek A.R., McKenzie C.I., Robinson M.J., Ding Z., Pitt C., O’Donnell K., Zotos D., Brink R., Tarlinton D.M., Quast I. (2022). IL-21 has a critical role in establishing germinal centers by amplifying early B cell proliferation. EMBO Rep..

[79] Samuels J., Ng Y.S., Coupillaud C., Paget D., Meffre E. (2005). Impaired early B cell tolerance in patients with rheumatoid arthritis. J. Exp. Med..

[80] Sorensen T.L., Trebst C., Kivisakk P., Klaege K.L., Majmudar A., Ravid R., Lassmann H., Olsen D.B., Strieter R.M., Ransohoff R.M. (2002). Multiple sclerosis: A study of CXCL10 and CXCR3 co-localization in the inflamed central nervous system. J. Neuroimmunol..

[81] Bogers L., Engelenburg H.J., Janssen M., Unger P.A., Melief M.J., Wierenga-Wolf A.F., Hsiao C.C., Mason M.R.J., Hamann J., van Langelaar J. (2023). Selective emergence of antibody-secreting cells in the multiple sclerosis brain. EBioMedicine.

[82] McGinley A.M., Sutton C.E., Edwards S.C., Leane C.M., DeCourcey J., Teijeiro A., Hamilton J.A., Boon L., Djouder N., Mills K.H.G. (2020). Interleukin-17A Serves a Priming Role in Autoimmunity by Recruiting IL-1beta-Producing Myeloid Cells that Promote Pathogenic T Cells. Immunity.

[83] Gijbels K., Proost P., Masure S., Carton H., Billiau A., Opdenakker G. (1993). Gelatinase B is present in the cerebrospinal fluid during experimental autoimmune encephalomyelitis and cleaves myelin basic protein. J. Neurosci. Res..

[84] McColl S.R., Staykova M.A., Wozniak A., Fordham S., Bruce J., Willenborg D.O. (1998). Treatment with anti-granulocyte antibodies inhibits the effector phase of experimental autoimmune encephalomyelitis. J. Immunol..

[85] McQualter J.L., Darwiche R., Ewing C., Onuki M., Kay T.W., Hamilton J.A., Reid H.H., Bernard C.C. (2001). Granulocyte macrophage colony-stimulating factor: A new putative therapeutic target in multiple sclerosis. J. Exp. Med..

[86] Carlson T., Kroenke M., Rao P., Lane T.E., Segal B. (2008). The Th17-ELR+ CXC chemokine pathway is essential for the development of central nervous system autoimmune disease. J. Exp. Med..

[87] Naegele M., Tillack K., Reinhardt S., Schippling S., Martin R., Sospedra M. (2012). Neutrophils in multiple sclerosis are characterized by a primed phenotype. J. Neuroimmunol..

[88] Flachenecker P. (2006). Epidemiology of neuroimmunological diseases. J. Neurol..

[89] Rosati G. (2001). The prevalence of multiple sclerosis in the world: An update. Neurol. Sci..

[90] Ebers G.C. (2008). Environmental factors and multiple sclerosis. Lancet Neurol..

[91] Kampman M., Wilsgaard T., Mellgren S. (2007). Outdoor activities and diet in childhood and adolescence relate to MS risk above the Arctic Circle. J. Neurol..

[92] Lauer K. (1997). Ecologic studies of multiple sclerosis. Neurology.

[93] Holick M.F. (2002). Vitamin D: The underappreciated D-lightful hormone that is important for skeletal and cellular health. Curr. Opin. Endocrinol. Diabetes Obes..

[94] McGrath J.J., Féron F.P., Burne T.H., Mackay-Sim A., Eyles D.W. (2004). Vitamin D3—Implications for brain development. J. Steroid Biochem. Mol. Biol..

[95] Cantarel B.L., Waubant E., Chehoud C., Kuczynski J., DeSantis T.Z., Warrington J., Venkatesan A., Fraser C.M., Mowry E.M. (2015). Gut microbiota in multiple sclerosis: Possible influence of immunomodulators. J. Investig. Med..

[96] Derrien M., Van Baarlen P., Hooiveld G., Norin E., Müller M., de Vos W.M. (2011). Modulation of mucosal immune response, tolerance, and proliferation in mice colonized by the mucin-degrader Akkermansia muciniphila. Front. Microbiol..

[97] Bauer H., Horowitz R.E., Levenson S.M., Popper H. (1963). The response of the lymphatic tissue to the microbial flora. Studies on germfree mice. Am. J. Pathol..

[98] Hapfelmeier S., Lawson M.A., Slack E., Kirundi J.K., Stoel M., Heikenwalder M., Cahenzli J., Velykoredko Y., Balmer M.L., Endt K. (2010). Reversible microbial colonization of germ-free mice reveals the dynamics of IgA immune responses. Science.

[99] Rescigno M., Urbano M., Valzasina B., Francolini M., Rotta G., Bonasio R., Granucci F., Kraehenbuhl J.P., Ricciardi-Castagnoli P. (2001). Dendritic cells express tight junction proteins and penetrate gut epithelial monolayers to sample bacteria. Nat. Immunol..

[100] Shan M., Gentile M., Yeiser J.R., Walland A.C., Bornstein V.U., Chen K., He B., Cassis L., Bigas A., Cols M. (2013). Mucus enhances gut homeostasis and oral tolerance by delivering immunoregulatory signals. Science.

[101] McDole J.R., Wheeler L.W., McDonald K.G., Wang B., Konjufca V., Knoop K.A., Newberry R.D., Miller M.J. (2012). Goblet cells deliver luminal antigen to CD103+ dendritic cells in the small intestine. Nature.

[102] Kedmi R., Najar T.A., Mesa K.R., Grayson A., Kroehling L., Hao Y., Hao S., Pokrovskii M., Xu M., Talbot J. (2022). A RORγt+ cell instructs gut microbiota-specific Treg cell differentiation. Nature.

[103] Xu M., Pokrovskii M., Ding Y., Yi R., Au C., Harrison O.J., Galan C., Belkaid Y., Bonneau R., Littman D.R. (2018). c-MAF-dependent regulatory T cells mediate immunological tolerance to a gut pathobiont. Nature.

[104] Mazmanian S.K., Liu C.H., Tzianabos A.O., Kasper D.L. (2005). An immunomodulatory molecule of symbiotic bacteria directs maturation of the host immune system. Cell.

[105] Spindler M.P., Siu S., Mogno I., Li Z., Yang C., Mehandru S., Britton G.J., Faith J.J. (2022). Human gut microbiota stimulate defined innate immune responses that vary from phylum to strain. Cell Host Microbe.

[106] Lee J.-Y., Hall J.A., Kroehling L., Wu L., Najar T., Nguyen H.H., Lin W.-Y., Yeung S.T., Silva H.M., Li D. (2020). Serum amyloid A proteins induce pathogenic Th17 cells and promote inflammatory disease. Cell.

[107] Ladinsky M.S., Araujo L.P., Zhang X., Veltri J., Galan-Diez M., Soualhi S., Lee C., Irie K., Pinker E.Y., Narushima S. (2019). Endocytosis of commensal antigens by intestinal epithelial cells regulates mucosal T cell homeostasis. Science.

[108] Nevo S., Kadouri N., Abramson J. (2019). Tuft cells: From the mucosa to the thymus. Immunol. Lett..

[109] Zwarycz B., Gracz A.D., Rivera K.R., Williamson I.A., Samsa L.A., Starmer J., Daniele M.A., Salter-Cid L., Zhao Q., Magness S.T. (2019). IL22 Inhibits Epithelial Stem Cell Expansion in an Ileal Organoid Model. Cell Mol. Gastroenterol. Hepatol..

[110] Tang W.W., Wang Z., Kennedy D.J., Wu Y., Buffa J.A., Agatisa-Boyle B., Li X.S., Levison B.S., Hazen S.L. (2015). Gut microbiota-dependent trimethylamine N-oxide (TMAO) pathway contributes to both development of renal insufficiency and mortality risk in chronic kidney disease. Circ. Res..

[111] Wang Z., Klipfell E., Bennett B.J., Koeth R., Levison B.S., DuGar B., Feldstein A.E., Britt E.B., Fu X., Chung Y.-M. (2011). Gut flora metabolism of phosphatidylcholine promotes cardiovascular disease. Nature.

[112] Wang H., Rong X., Zhao G., Zhou Y., Xiao Y., Ma D., Jin X., Wu Y., Yan Y., Yang H. (2022). The microbial metabolite trimethylamine N-oxide promotes antitumor immunity in triple-negative breast cancer. Cell Metab..

[113] Kelly C.J., Zheng L., Campbell E.L., Saeedi B., Scholz C.C., Bayless A.J., Wilson K.E., Glover L.E., Kominsky D.J., Magnuson A. (2015). Crosstalk between Microbiota-Derived Short-Chain Fatty Acids and Intestinal Epithelial HIF Augments Tissue Barrier Function. Cell Host Microbe.

[114] Liu L., Li L., Min J., Wang J., Wu H., Zeng Y., Chen S., Chu Z. (2012). Butyrate interferes with the differentiation and function of human monocyte-derived dendritic cells. Cell Immunol..

[115] Lee C., Kim B.G., Kim J.H., Chun J., Im J.P., Kim J.S. (2017). Sodium butyrate inhibits the NF-kappa B signaling pathway and histone deacetylation, and attenuates experimental colitis in an IL-10 independent manner. Int. Immunopharmacol..

[116] Bansal T., Alaniz R.C., Wood T.K., Jayaraman A. (2010). The bacterial signal indole increases epithelial-cell tight-junction resistance and attenuates indicators of inflammation. Proc. Natl. Acad. Sci. USA.

[117] Wu K., Yuan Y., Yu H., Dai X., Wang S., Sun Z., Wang F., Fei H., Lin Q., Jiang H. (2020). The gut microbial metabolite trimethylamine N-oxide aggravates GVHD by inducing M1 macrophage polarization in mice. Blood J. Am. Soc. Hematol..

[118] Chen M.l., Zhu X.h., Ran L., Lang H.d., Yi L., Mi M.t. (2017). Trimethylamine-N-Oxide induces vascular inflammation by activating the NLRP3 inflammasome through the SIRT3-SOD2-mtROS signaling pathway. J. Am. Heart Assoc..

[119] Mirji G., Worth A., Bhat S.A., El Sayed M., Kannan T., Goldman A.R., Tang H.-Y., Liu Q., Auslander N., Dang C.V. (2022). The microbiome-derived metabolite TMAO drives immune activation and boosts responses to immune checkpoint blockade in pancreatic cancer. Sci. Immunol..

[120] Leidy J. (1853). A Flora and Fauna Within Living Animals.

[121] Ursell L.K., Metcalf J.L., Parfrey L.W., Knight R. (2012). Defining the human microbiome. Nutr. Rev..

[122] Nissle A. (1916). Über die Grundlagen einer neuen ursächlichen Bekämpfung der pathologischen Darmflora. Dtsch. Med. Wochenschr..

[123] Andersson A.F., Lindberg M., Jakobsson H., Backhed F., Nyren P., Engstrand L. (2008). Comparative analysis of human gut microbiota by barcoded pyrosequencing. PLoS ONE.

[124] Belizario J.E., Napolitano M. (2015). Human microbiomes and their roles in dysbiosis, common diseases, and novel therapeutic approaches. Front. Microbiol..

[125] Rinninella E., Raoul P., Cintoni M., Franceschi F., Miggiano G.A.D., Gasbarrini A., Mele M.C. (2019). What is the Healthy Gut Microbiota Composition? A Changing Ecosystem across Age, Environment, Diet, and Diseases. Microorganisms.

[126] Pei Z., Bini E.J., Yang L., Zhou M., Francois F., Blaser M.J. (2004). Bacterial biota in the human distal esophagus. Proc. Natl. Acad. Sci. USA.

[127] Nikitina D., Lehr K., Vilchez-Vargas R., Jonaitis L.V., Urba M., Kupcinskas J., Skieceviciene J., Link A. (2023). Comparison of genomic and transcriptional microbiome analysis in gastric cancer patients and healthy individuals. World J. Gastroenterol..

[128] King C.H., Desai H., Sylvetsky A.C., LoTempio J., Ayanyan S., Carrie J., Crandall K.A., Fochtman B.C., Gasparyan L., Gulzar N. (2019). Baseline human gut microbiota profile in healthy people and standard reporting template. PLoS ONE.

[129] Vuik F., Dicksved J., Lam S.Y., Fuhler G.M., van der Laan L., van de Winkel A., Konstantinov S.R., Spaander M., Peppelenbosch M.P., Engstrand L. (2019). Composition of the mucosa-associated microbiota along the entire gastrointestinal tract of human individuals. United Eur. Gastroenterol. J..

[130] Korpela K., Blakstad E.W., Moltu S.J., Strommen K., Nakstad B., Ronnestad A.E., Braekke K., Iversen P.O., Drevon C.A., de Vos W. (2018). Intestinal microbiota development and gestational age in preterm neonates. Sci. Rep..

[131] Rotimi V.O., Duerden B.I. (1981). The development of the bacterial flora in normal neonates. J. Med. Microbiol..

[132] Jakobsson H.E., Abrahamsson T.R., Jenmalm M.C., Harris K., Quince C., Jernberg C., Bjorksten B., Engstrand L., Andersson A.F. (2014). Decreased gut microbiota diversity, delayed Bacteroidetes colonisation and reduced Th1 responses in infants delivered by caesarean section. Gut.

[133] Tomkins A.M., Bradley A.K., Oswald S., Drasar B.S. (1981). Diet and the faecal microflora of infants, children and adults in rural Nigeria and urban U.K. J. Hyg..

[134] Zhang D., Yang X., Li D., Li L., Zhang D., Peng Y. (2019). Effects of different modes of delivery and feeding on intestinal flora of newborns and infants with different ages. Iran. J. Pediatr..

[135] Sonnenburg E.D., Smits S.A., Tikhonov M., Higginbottom S.K., Wingreen N.S., Sonnenburg J.L. (2016). Diet-induced extinctions in the gut microbiota compound over generations. Nature.

[136] Kaczmarek J.L., Liu X., Charron C.S., Novotny J.A., Jeffery E.H., Seifried H.E., Ross S.A., Miller M.J., Swanson K.S., Holscher H.D. (2019). Broccoli consumption affects the human gastrointestinal microbiota. J. Nutr. Biochem..

[137] Holscher H.D., Guetterman H.M., Swanson K.S., An R., Matthan N.R., Lichtenstein A.H., Novotny J.A., Baer D.J. (2018). Walnut Consumption Alters the Gastrointestinal Microbiota, Microbially Derived Secondary Bile Acids, and Health Markers in Healthy Adults: A Randomized Controlled Trial. J. Nutr..

[138] Schwiertz A., Taras D., Schafer K., Beijer S., Bos N.A., Donus C., Hardt P.D. (2010). Microbiota and SCFA in lean and overweight healthy subjects. Obesity.

[139] Ley R.E., Turnbaugh P.J., Klein S., Gordon J.I. (2006). Human gut microbes associated with obesity. Nature.

[140] Giongo A., Gano K.A., Crabb D.B., Mukherjee N., Novelo L.L., Casella G., Drew J.C., Ilonen J., Knip M., Hyoty H. (2011). Toward defining the autoimmune microbiome for type 1 diabetes. ISME J..

[141] Frank D.N., St Amand A.L., Feldman R.A., Boedeker E.C., Harpaz N., Pace N.R. (2007). Molecular-phylogenetic characterization of microbial community imbalances in human inflammatory bowel diseases. Proc. Natl. Acad. Sci. USA.

[142] Hevia A., Milani C., Lopez P., Cuervo A., Arboleya S., Duranti S., Turroni F., Gonzalez S., Suarez A., Gueimonde M. (2014). Intestinal dysbiosis associated with systemic lupus erythematosus. mBio.

[143] Zhang X., Zhang D., Jia H., Feng Q., Wang D., Liang D., Wu X., Li J., Tang L., Li Y. (2015). The oral and gut microbiomes are perturbed in rheumatoid arthritis and partly normalized after treatment. Nat. Med..

[144] Zheng P., Zeng B., Zhou C., Liu M., Fang Z., Xu X., Zeng L., Chen J., Fan S., Du X. (2016). Gut microbiome remodeling induces depressive-like behaviors through a pathway mediated by the host’s metabolism. Mol. Psychiatry.

[145] Huang B., Chau S.W.H., Liu Y., Chan J.W.Y., Wang J., Ma S.L., Zhang J., Chan P.K.S., Yeoh Y.K., Chen Z. (2023). Gut microbiome dysbiosis across early Parkinson’s disease, REM sleep behavior disorder and their first-degree relatives. Nat. Commun..

[146] Boertien J.M., Murtomaki K., Pereira P.A.B., van der Zee S., Mertsalmi T.H., Levo R., Nojonen T., Makinen E., Jaakkola E., Laine P. (2022). Fecal microbiome alterations in treatment-naive de novo Parkinson’s disease. NPJ Park. Dis..

[147] Jemimah S., Chabib C.M.M., Hadjileontiadis L., AlShehhi A. (2023). Gut microbiome dysbiosis in Alzheimer’s disease and mild cognitive impairment: A systematic review and meta-analysis. PLoS ONE.

[148] Chen J., Chia N., Kalari K.R., Yao J.Z., Novotna M., Paz Soldan M.M., Luckey D.H., Marietta E.V., Jeraldo P.R., Chen X. (2016). Multiple sclerosis patients have a distinct gut microbiota compared to healthy controls. Sci. Rep..

[149] Miyake S., Kim S., Suda W., Oshima K., Nakamura M., Matsuoka T., Chihara N., Tomita A., Sato W., Kim S.W. (2015). Dysbiosis in the Gut Microbiota of Patients with Multiple Sclerosis, with a Striking Depletion of Species Belonging to Clostridia XIVa and IV Clusters. PLoS ONE.

[150] Jangi S., Gandhi R., Cox L.M., Li N., von Glehn F., Yan R., Patel B., Mazzola M.A., Liu S., Glanz B.L. (2016). Alterations of the human gut microbiome in multiple sclerosis. Nat. Commun..

[151] Laaker C., Hsu M., Fabry Z., Miller S.D., Karpus W.J. (2021). Experimental Autoimmune Encephalomyelitis in the Mouse. Curr. Protoc..

[152] Berer K., Mues M., Koutrolos M., Rasbi Z.A., Boziki M., Johner C., Wekerle H., Krishnamoorthy G. (2011). Commensal microbiota and myelin autoantigen cooperate to trigger autoimmune demyelination. Nature.

[153] Berer K., Gerdes L.A., Cekanaviciute E., Jia X., Xiao L., Xia Z., Liu C., Klotz L., Stauffer U., Baranzini S.E. (2017). Gut microbiota from multiple sclerosis patients enables spontaneous autoimmune encephalomyelitis in mice. Proc. Natl. Acad. Sci. USA.

[154] Cekanaviciute E., Yoo B.B., Runia T.F., Debelius J.W., Singh S., Nelson C.A., Kanner R., Bencosme Y., Lee Y.K., Hauser S.L. (2017). Gut bacteria from multiple sclerosis patients modulate human T cells and exacerbate symptoms in mouse models. Proc. Natl. Acad. Sci. USA.

[155] Cosorich I., Dalla-Costa G., Sorini C., Ferrarese R., Messina M.J., Dolpady J., Radice E., Mariani A., Testoni P.A., Canducci F. (2017). High frequency of intestinal T(H)17 cells correlates with microbiota alterations and disease activity in multiple sclerosis. Sci. Adv..

[156] Engen S.A., Valen Rukke H., Becattini S., Jarrossay D., Blix I.J., Petersen F.C., Sallusto F., Schenck K. (2014). The oral commensal Streptococcus mitis shows a mixed memory Th cell signature that is similar to and cross-reactive with Streptococcus pneumoniae. PLoS ONE.

[157] Sokol H., Pigneur B., Watterlot L., Lakhdari O., Bermúdez-Humarán L.G., Gratadoux J.-J., Blugeon S., Bridonneau C., Furet J.-P., Corthier G. (2008). Faecalibacterium prausnitzii is an anti-inflammatory commensal bacterium identified by gut microbiota analysis of Crohn disease patients. Proc. Natl. Acad. Sci. USA.

[158] Pryde S.E., Duncan S.H., Hold G.L., Stewart C.S., Flint H.J. (2002). The microbiology of butyrate formation in the human colon. FEMS Microbiol. Lett..

[159] Siaw Y., Hart A. (2013). Commentary: Is F aecalibacterium prausnitzii a potential treatment for maintaining remission in ulcerative colitis?. Aliment. Pharmacol. Ther..

[160] Macfarlane S., Macfarlane G.T. (2006). Composition and metabolic activities of bacterial biofilms colonizing food residues in the human gut. Appl. Environ. Microbiol..

[161] Tedelind S., Westberg F., Kjerrulf M., Vidal A. (2007). Anti-inflammatory properties of the short-chain fatty acids acetate and propionate: A study with relevance to inflammatory bowel disease. World J. Gastroenterol..

[162] Arpaia N., Campbell C., Fan X., Dikiy S., van der Veeken J., deRoos P., Liu H., Cross J.R., Pfeffer K., Coffer P.J. (2013). Metabolites produced by commensal bacteria promote peripheral regulatory T-cell generation. Nature.

[163] Haghikia A., Jorg S., Duscha A., Berg J., Manzel A., Waschbisch A., Hammer A., Lee D.H., May C., Wilck N. (2015). Dietary Fatty Acids Directly Impact Central Nervous System Autoimmunity via the Small Intestine. Immunity.

[164] Farrokhi V., Nemati R., Nichols F.C., Yao X., Anstadt E., Fujiwara M., Grady J., Wakefield D., Castro W., Donaldson J. (2013). Bacterial lipodipeptide, Lipid 654, is a microbiome-associated biomarker for multiple sclerosis. Clin. Transl. Immunol..

[165] Clark R.B., Cervantes J.L., Maciejewski M.W., Farrokhi V., Nemati R., Yao X., Anstadt E., Fujiwara M., Wright K.T., Riddle C. (2013). Serine lipids of Porphyromonas gingivalis are human and mouse Toll-like receptor 2 ligands. Infect. Immun..

[166] Brown J., Everett C., Barragan J.A., Vargas-Medrano J., Gadad B.S., Nichols F., Cervantes J.L. (2022). Multiple Sclerosis-associated Bacterial Ligand 654. Arch. Med. Res..

[167] Jantaratnotai N., Utaisincharoen P., Sanvarinda P., Thampithak A., Sanvarinda Y. (2013). Phytoestrogens mediated anti-inflammatory effect through suppression of IRF-1 and pSTAT1 expressions in lipopolysaccharide-activated microglia. Int. Immunopharmacol..

[168] Kadowaki A., Saga R., Lin Y., Sato W., Yamamura T. (2019). Gut microbiota-dependent CCR9^+^CD4^+^ T cells are altered in secondary progressive multiple sclerosis. Brain.

[169] Guy-Grand D., Vassalli P., Eberl G., Pereira P., Burlen-Defranoux O., Lemaitre F., Di Santo J.P., Freitas A.A., Cumano A., Bandeira A. (2013). Origin, trafficking, and intraepithelial fate of gut-tropic T cells. J. Exp. Med..

[170] Kadowaki A., Miyake S., Saga R., Chiba A., Mochizuki H., Yamamura T. (2016). Gut environment-induced intraepithelial autoreactive CD4^+^ T cells suppress central nervous system autoimmunity via LAG-3. Nat. Commun..

[171] Song J.Y., Larson N.R., Thati S., Torres-Vazquez I., Martinez-Rivera N., Subelzu N.J., Leon M.A., Rosa-Molinar E., Schoneich C., Forrest M.L. (2019). Glatiramer acetate persists at the injection site and draining lymph nodes via electrostatically-induced aggregation. J. Control. Release.

[172] Messina S., Patti F. (2013). The pharmacokinetics of glatiramer acetate for multiple sclerosis treatment. Expert. Opin. Drug Metab. Toxicol..

[173] McKeage K. (2015). Glatiramer Acetate 40 mg/mL in Relapsing-Remitting Multiple Sclerosis: A Review. CNS Drugs.

[174] van den Hoogen W.J., Laman J.D., ‘t Hart B.A. (2017). Modulation of multiple sclerosis and its animal model experimental autoimmune encephalomyelitis by food and gut microbiota. Front. Immunol..

[175] Ochoa-Repáraz J., Mielcarz D.W., Ditrio L.E., Burroughs A.R., Foureau D.M., Haque-Begum S., Kasper L.H. (2009). Role of gut commensal microflora in the development of experimental autoimmune encephalomyelitis. J. Immunol..

[176] Ochoa-Repáraz J., Mielcarz D.W., Haque-Begum S., Kasper L.H. (2010). Induction of a regulatory B cell population in experimental allergic encephalomyelitis by alteration of the gut commensal microflora. Gut Microbes.

[177] Planas R., Santos R., Tomas-Ojer P., Cruciani C., Lutterotti A., Faigle W., Schaeren-Wiemers N., Espejo C., Eixarch H., Pinilla C. (2018). GDP-l-fucose synthase is a CD4^+^ T cell-specific autoantigen in DRB3*02:02 patients with multiple sclerosis. Sci. Transl. Med..

[178] Ma B., Simala-Grant J.L., Taylor D.E. (2006). Fucosylation in prokaryotes and eukaryotes. Glycobiology.

[179] Hirota K., Kanitani H., Nemoto K., Ono T., Miyake Y. (1995). Cross-reactivity between human sialyl Lewis(x) oligosaccharide and common causative oral bacteria of infective endocarditis. FEMS Immunol. Med. Microbiol..

[180] Lee H.S., Choe G., Kim W.H., Kim H.H., Song J., Park K.U. (2006). Expression of Lewis antigens and their precursors in gastric mucosa: Relationship with Helicobacter pylori infection and gastric carcinogenesis. J. Pathol..

[181] Yokota S., Amano K., Hayashi S., Kubota T., Fujii N., Yokochi T. (1998). Human antibody response to Helicobacter pylori lipopolysaccharide: Presence of an immunodominant epitope in the polysaccharide chain of lipopolysaccharide. Infect. Immun..

[182] Garcia-Vallejo J.J., Ilarregui J.M., Kalay H., Chamorro S., Koning N., Unger W.W., Ambrosini M., Montserrat V., Fernandes R.J., Bruijns S.C. (2014). CNS myelin induces regulatory functions of DC-SIGN-expressing, antigen-presenting cells via cognate interaction with MOG. J. Exp. Med..

[183] Lee J., Ha S., Kim M., Kim S.W., Yun J., Ozcan S., Hwang H., Ji I.J., Yin D., Webster M.J. (2020). Spatial and temporal diversity of glycome expression in mammalian brain. Proc. Natl. Acad. Sci. USA.

[184] GBD 2016 Multiple Sclerosis Collaborators (2019). Global, regional, and national burden of multiple sclerosis 1990-2016: A systematic analysis for the Global Burden of Disease Study 2016. Lancet Neurol..

[185] Janardhan V., Bakshi R. (2002). Quality of life in patients with multiple sclerosis: The impact of fatigue and depression. J. Neurol. Sci..

[186] Sumelahti M.L., Tienari P.J., Wikstrom J., Palo J., Hakama M. (2001). Increasing prevalence of multiple sclerosis in Finland. Acta Neurol. Scand..

[187] Barnett M.H., Williams D.B., Day S., Macaskill P., McLeod J.G. (2003). Progressive increase in incidence and prevalence of multiple sclerosis in Newcastle, Australia: A 35-year study. J. Neurol. Sci..

[188] Ye P., Garvey P.B., Zhang P., Nelson S., Bagby G., Summer W.R., Schwarzenberger P., Shellito J.E., Kolls J.K. (2001). Interleukin-17 and lung host defense against Klebsiella pneumoniae infection. Am. J. Respir. Cell Mol. Biol..

[189] Schnyder-Candrian S., Togbe D., Couillin I., Mercier I., Brombacher F., Quesniaux V., Fossiez F., Ryffel B., Schnyder B. (2006). Interleukin-17 is a negative regulator of established allergic asthma. J. Exp. Med..

[190] Lee J.S., Tato C.M., Joyce-Shaikh B., Gulen M.F., Cayatte C., Chen Y., Blumenschein W.M., Judo M., Ayanoglu G., McClanahan T.K. (2015). Interleukin-23-Independent IL-17 Production Regulates Intestinal Epithelial Permeability. Immunity.

[191] Drekonja D., Reich J., Gezahegn S., Greer N., Shaukat A., MacDonald R., Rutks I., Wilt T.J. (2015). Fecal Microbiota Transplantation for Clostridium difficile Infection: A Systematic Review. Ann. Intern. Med..

[192] Varga A., Kocsis B., Sipos D., Kasa P., Vigvari S., Pal S., Dembrovszky F., Farkas K., Peterfi Z. (2021). How to Apply FMT More Effectively, Conveniently and Flexible—A Comparison of FMT Methods. Front. Cell Infect. Microbiol..

[193] Vigvari S., Nemes Z., Vincze A., Solt J., Sipos D., Feiszt Z., Kovacs B., Bartos B., Peterfi Z. (2015). Faecal microbiota transplantation in Clostridium difficile infections. Infect. Dis..

[194] Al K.F., Craven L.J., Gibbons S., Parvathy S.N., Wing A.C., Graf C., Parham K.A., Kerfoot S.M., Wilcox H., Burton J.P. (2022). Fecal microbiota transplantation is safe and tolerable in patients with multiple sclerosis: A pilot randomized controlled trial. Mult. Scler. J. Exp. Transl. Clin..

